# A Comparison of the Analgesia Efficacy and Side Effects of Paravertebral Compared with Epidural Blockade for Thoracotomy: An Updated Meta-Analysis

**DOI:** 10.1371/journal.pone.0096233

**Published:** 2014-05-05

**Authors:** Xibing Ding, Shuqing Jin, Xiaoyin Niu, Hao Ren, Shukun Fu, Quan Li

**Affiliations:** 1 Department of Anesthesiology, Shanghai Tenth People's Hospital, Tongji University School of Medicine, Shanghai, China; 2 Department of Anesthesiology, East Hospital, Tongji University School of Medicine, Shanghai, China; San Raffaele Scientific Institute, Italy

## Abstract

**Objective:**

The most recent systematic review and meta-analysis comparing the analgesic efficacy and side effects of paravertebral and epidural blockade for thoracotomy was published in 2006. Nine well-designed randomized trials with controversial results have been published since then. The present report constitutes an updated meta-analysis of this issue.

**Summary of Background:**

Thoracotomy is a major surgical procedure and is associated with severe postoperative pain. Epidural analgesia is the gold standard for post-thoracotomy pain management, but has its limitations and contraindications, and paravertebral blockade is increasingly popular. However, it has not been decided whether the analgesic effect of the two methods is comparable, or whether paravertebral blockade leads to a lower incidence of adverse side effects after thoracotomy.

**Methods:**

Two reviewers independently searched the databases PubMed, EMBASE, and the Cochrane Library (last performed on 1 February, 2013) for reports of studies comparing post-thoracotomy epidural analgesia and paravertebral blockade. The same individuals independently extracted data from the appropriate studies.

**Result:**

Eighteen trials involving 777 patients were included in the current analysis. There was no significant difference in pain scores between paravertebral blockade and epidural analgesia at 4–8, 24, 48 hours, and the rates of pulmonary complications and morphine usage during the first 24 hours were also similar. However, paravertebral blockade was better than epidural analgesia in reducing the incidence of urinary retention (*p*<0.0001), nausea and vomiting (*p* = 0.01), hypotension (*p*<0.00001), and rates of failed block were lower in the paravertebral blockade group (*p* = 0.01).

**Conclusions:**

This meta-analysis showed that PVB can provide comparable pain relief to traditional EPI, and may have a better side-effect profile for pain relief after thoracic surgery. Further high-powered randomized trials are to need to determine whether PVB truly offers any advantages over EPI.

## Introduction

Thoracotomy, the surgical incision of the pleural cavity or chest wall, induces severe postoperative pain [1]. The pain can cause respiratory complications such as hypoxia (inadequate oxygen), atelectasis (lung collapse) and pulmonary infection due to shallow breathing and impaired coughing. If severe enough, the postoperative pain can lead to dreadful respiratory disorders including respiratory failure and other complications [2].

In addition, chronic pain after thoracotomy is common and may continue for many years, especially in patients who experienced acute post-operative pain [3], [4]. However, adequate postoperative analgesia facilitates recovery [5].

Regional anesthesia may reduce the rate of chronic pain after surgery [6]. Although epidural analgesia is clearly effective for managing postoperative pain after thoracotomy, it still has limitations and contraindications. For instance, the number of patients using antiplatelet agents such as aspirin and clopidogrel are considerably more than before. The failure rate of epidural analgesia has been reported to be as high as 12% [7]. Epidural analgesia also carries the risk for severe complications such as epidural abscess and spinal hematoma [7]. Paravertebral analgesia has been studied as a possible alternative to epidural analgesia for thoracotomy. Because the analgesic effects of paravertebral blockade (PVB) are comparable to epidural analgesia (EPI), PVB may avoid the risks of EPI such as hypotension and urinary retention [8], and catheterization for PVB can be placed under direct vision during the surgery.

Davies et al. [9] reported a systematic review and meta-analysis of 10 randomized trials comparing PVB with EPI. They found that PVB and epidural analgesia provide comparable pain relief after thoracotomy, but PVB had a better side-effect profile and fewer pulmonary complications. However, recent various trials have achieved different results [10]–[18]. The current study is an updated meta-analysis comparing the efficacy and adverse effects of PVB and EPI in preventing pain associated with thoracotomy.

## Methods

### Search strategy

We identified randomized controlled trials by electronically searching the databases: Pubmed, EMBASE, and the Cochrane Library for reports published from 1 January 2006 to 2 February 2013. The following medical subject headings were included: paravertebral, epidural, thoracotomy, and randomized controlled trial. Alternative spellings were considered when searching. We removed duplicates that were identified in multiple database searches.

### Inclusion criteria

Randomized controlled trials that compared the analgesic efficacy and side effects of PVB and EPI for thoracotomy were included. Studies published only in English were included. The dosages and other details of anesthesia drug administration were not limited. Only studies concerning thoracotomy were allowed and trials regarding breast cancer, and lumbar epidural block were excluded.

### Selection of studies

Two reviewers (Xibing Ding, Shuqing Jin) used the pre-specified criteria to screen for relevant titles, abstracts, and full papers. An article was removed if it did not meet the inclusion criteria. If these reviewers reached different final selection decisions, a third reviewer (Quan Li, Shukun Fu) was consulted.

### Date extraction

We extracted the following data from the included articles: First author; publishing date; number of patients; study design; description of interventions between PVB and EPI group; postoperative visual analogue scale (VAS) scores at 4–8, 24, and 48 h; morphine usage during the first 24 h; and pulmonary complications, urinary retention, nausea and vomiting, hypotension and failed rate of block. The definitions of the above indicators conformed to those of the original authors. As the primary outcomes, we defined the analgesic effect in terms of VAS scores at postoperative 4–8 h, 24 h, 48 h, and morphine usage during the first 24 h. Secondary outcomes were the remaining pulmonary complications and urinary retention. These data were then compiled into a standard table. The two reviewers (Xibing Ding, Shuqing Jin) who selected the appropriate studies also extracted the data and evaluated the risk of bias. An arbiter (Quan Li) was consulted to reconcile any disagreement.

### Assessing the risk of bias

We used the Cochrane Handbook V5.0.2 [19] to assess the risk of bias for all articles. The following information was evaluated: random sequence generation, allocation concealment, blinding, incomplete outcome data, selective reporting, and other bias. Two reviewers (Xiaoyin Niu, Hao Ren) evaluated the methodological quality of all articles. An arbiter (Quan Li) was consulted to reconcile any disagreements.

### Statistical analysis

Review Manager Software (Revman 5.0, Cochrane Collaboration, Oxford, United Kingdom) was used for the meta-analysis. Heterogeneity among the studies was evaluated using the I^2^ statistic and chi-squared test. A fixed effects model was used if the heterogeneity test did not reveal a statistical significance (I^2^<50%, *p*>0.1). Otherwise, we adopted the random effects model. For the continuous variables in the studies included in this meta-analysis (VAS score at postoperative 4–8, 24 and 48 h, and morphine usage at 24 h), used mean difference (MD) and 95% confidence interval (95% CI). For dichotomous variables (pulmonary complications, urinary retention, nausea and vomiting, hypotension, and failed rates of blockage), we used the odds ratio (OR) and 95% CI. All tests of statistical significance were two-sided [20]. If the heterogeneity was>50%, we performed a sensitivity analysis by sequentially removing each study and reanalyzing the remaining dataset. Also, we analyzed only data that had a low risk of bias.

## Results

### Search results

**Figure 1:**
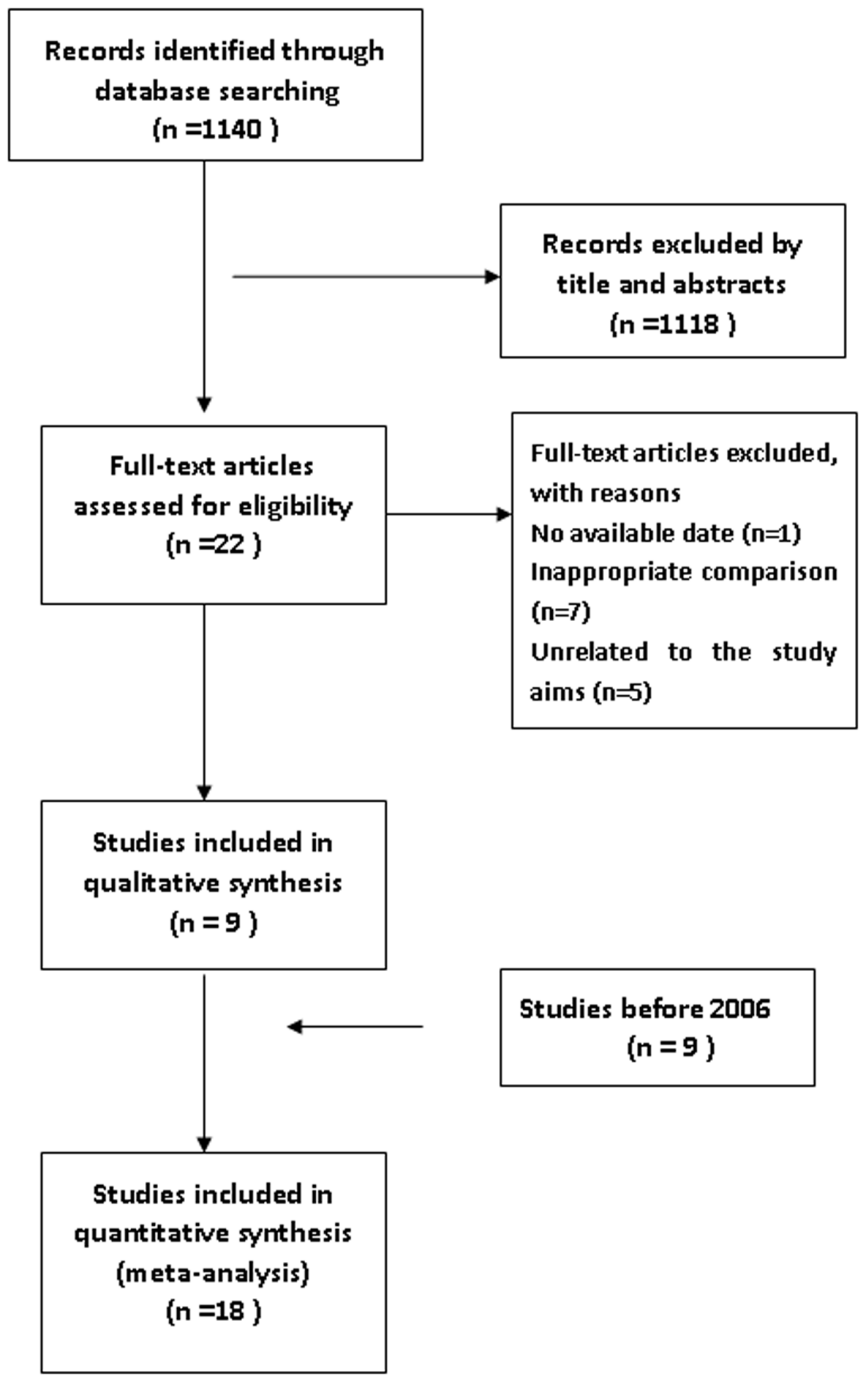
Flow chart of study selection.

Initially, 1330 records were identified through the PubMed, EMBASE, and Cochrane Library database (Fig. 1). Of these, 22 potentially eligible articles, only 9 were found to fulfill the inclusion criteria [10]–[18]. The remaining 13 article [21]–[33] were removed because the trials did not compare PVB and EPI, or the original data were not available from the authors, or the original data was not relevant to the aims of our study. We just included 9 articles from Davies et al. [9], because the results of Wedad et al. included in Davies et al. meta-analysis had no effect on the updated research. Therefore, 18 studies [10]–[18], [34]–[42] comprising 777 patients were included in the present meta-analysis (Table 1). A detailed explanation of the full electronic search strategy for Pubmed is shown in Figure 1. A detailed explanation of the search strategy for the Cochrane Library is shown in Appendix S1.

**Table 1:** Characteristics of included studies.

Article	Type of surgery	Number of patients	PVB Group	EPI Group
Kunihisa et al 2011	Thoracotomy	48	5 ml of 0.75% ropivacaine a bolus dose followed by a 2nd bolus of 5 ml of 0.75% ropivacaine. Then continuous infusion of 0.2% ropivacaine at 4 ml/h over a period of 60 hours.	A continuous infusion of 0.2% ropivacaine at 4 ml/h was started at the end of surgery after the injection of a 2nd bolus of 5 ml of 0.75% ropivacaine and continued for 60 hours
Jay S et al 2012	Thoracotomy	75	0.25% bupivacaine at 8 ml/h.	Basal 2 ml/h with 1 ml every 10 minutes via patient-controlled analgesia [PCA] were 0.25% bupivacaine alone or 0.25% bupivacaine with 0.01 mg/ml of hydromorphone,
A Casati et al 2006	Thoracotomy	42	15 ml of 0.75% ropivacaine divided into three injections at the T4, T5 and T6 levels (5 ml at each injection site)	5 ml of 0.75% ropivacaine
Mehta et al 2008	Thoracotomy	36	Bolus dose of 8 ml of 0.5% bupivacaine; infusion of 0.25% bupivacaine at the rate of 0.1 ml/kg/hr	Bolus dose of 8 ml of 0.5% bupivacaine; an infusion of 0.25% bupivacaine at the rate of 0.1 ml/kg/hr
Gultekin et al 2009	Thoracotomy	44	Infusion of 0.25% of bupivacaine at a rate of 0.10 ml/kg/1 h (1 h lock and 2 ml bolus) through patient-controlled elastomeric infusion pump	Bupivacaine (5 ml of 0.25%) at a rate of 0.10 ml/kg/1 h (1 h lock and 2 ml bolus) through a patient-controlled elastomeric infusion pump
Messinaa et al 2009	Thoracotomy	24	Infusion of 0.25% of bupivacaine at a rate of 0.10 ml/kg 1 h/1 (1 h lock and 2 ml bolus) through patient-controlled elastomeric infusion pump	Infusion of 0.25% of bupivacaine at a rate of 0.10 ml kg 1 h 1(1 h lock and 2 ml bolus) through patient-controlled elastomeric infusion pump
Tatjana et al 2011	Thoracotomy	32	Combination of 0.5% levobupivacaine and 30 Kg/kg morphine.	A mixture of 0.25% levobupivacaine with 30 Kg/kg morphine
Medha et al 2009	Thoracotomy	30	A bolus dose of bupivacaine 0.5% in a volume of 0.3 ml/kg (1.5 mg/kg) and a continuous infusion of bupivacaine 0.25% at a rate of 0.1 ml/kg/hr to 0.2 ml/kg/hr.	Bupivacaine 0.5% in a volume of 1 mL/segment to 1.5 ml/segment as bolus,then an infusion of bupivacaine 0.125% at a rate of 0.1 ml/kg/hr to 0.2 ml/kg/hr.
Ghassan et al 2012	Thoracotomy	42	A loading dose of 20 ml of 0.25% bupivacaine with 5 mg ml 1 of adrenaline,continuous infusion of 0.125% bupivacaine 8 ml/h/1 was started	10 ml of 0.125% bupivacaine wit 5 mg/ml of adrenaline, a continuous infusion of 0.125% bupivacaine 8 ml/h 1
Kaiser et al 1998	Thoracotomy	124	Pre-induction bupivacaine 0.24 bolus; intraoperative bupivacaine 0/5% bolus; postoprative bupivacaine 0.25% infusion	Bupivacaine 0.125%+morphine infusion
Richardson et al 1999	Thoracotomy	29	0.5% bupivacaine bolus infusion	Thoracic bupivacaine 0.5% bolus, then bupivacaine 0.125% infusion
Leaver et al 2006	Thoracotomy	50	Ropivacaine 0.475% bolus	Thoracic ropvacaine 0.2%+sufentanil bolus, then infusion
Matthews et al 1989	Thoracotomy	20	Bupivacaine 0.25% bolus+infusion	Thoracic bupivacaine 0.25% bolus, then infusion
De Cosmo et al 2002	Thoracotomy	20	Pre-induction bupivacaine 0.5% bolus; intraoperative bupivacaine 0.25% bolus; postoperative bupivacaine 0.5% infusion	Thoracic bupivacaine 0.25% bolus, then infusion
Perttunen et al 1995	Thoracotomy	40	Bupivacaine 0.25% bolus+infusion	Thoracic bupivacaine 0.26% bolus, then infusion
Dhole et al 2001	Thoracotomy	30	Bupivacaine 0.5% bolus+infusion	Thoracic bupivacaine 0.5% intraoperatively, then 0.25–0.375% bupivacaine+fentanyl infusion
Luketich et al 2005	Thoracotomy	41	Bupivacaine 0.5% bolus+bupivacaine 0.25% infusion	Thoracic bupivacaine 0.5% bolus, then bupivacaine 0.25% infusion
Bimston et al 1999	Thoracotomy	50	Bupivacaine 0.5% bolus+bupivacaine 0.25% infusion	Thoracic bupivacaine 0.5% bolus, then bupivacaine 0.25% infusion

Among the 18 included studies, the insertion methods for PVB varied. PVB was inserted before the surgery in some studies [34], [38] whether the catheter was inserted at the end of surgery in others. Furthermore, the kinds and concentrations of anesthesia drugs are also different. The different concentrations of local anesthetic (LA) were determined by standard for epidural (low LA concentration) and for paravertebral (high LA concentration) analgesia.

### Risk of bias of included studies

According to the Cochrane Handbook V5.0.2, each study had a high risk of bias (Table 2). Thus, the evidence of this meta-analysis has a high overall risk of bias. The authors of each study described it as randomized, but the randomization method was not specified in 8 studies. Six studies used the allocation concealment method. The participants of the allocated treatment could not be blinded because the blockade technique used for each was clinically evident, but those who adjudged outcomes were blinded in three trials. Incomplete outcome data were considered low risk of bias in all articles. Selecting reporting bias was considered ‘low’ for with no access to each trial's original protocol. Among random sequence generation, allocation concealment and blinding, only when any two of them are ‘low’, the overall risk of bias is considered as low.

**Table 2:** Risk of bias assessment of included studies.

Article	overall risk of bias	Random sequence generation	Allocation concealment	Blinding	Incomplete outcome data	Selective reporting	other bias
Kunihisa et al 2011	High	Low	unclear	high	low	low	high
Jay S et al 2012	Low	Low	low	low	low	low	high
A Casati et al 2006	Low	Low	low	low	low	low	high
Mehta et al 2008	high	unclear	unclear	high	low	low	high
Gultekin et al 2009	low	Low	low	high	low	low	high
Messinaa et al 2009	low	Low	low	high	low	low	high
Tatjana et al 2011	low	low	low	high	low	low	high
Medha et al 2009	low	Low	low	high	low	low	high
Ghassan et al 2012	low	Low	unclear	low	low	low	high
Kaiser et al 1998	high	unclear	unclear	high	low	low	high
Richardson et al 1999	high	Low	unclear	high	low	low	high
Leaver et al.2006	high	unclear	unclear	high	low	low	high
Matthews et al 1989	high	unclear	unclear	high	low	low	high
De Cosmo et al 2002	high	Low	unclear	high	low	low	high
Perttunen et al 1995	high	unclear	unclear	high	low	low	high
Dhole et al 2001	high	unclear	unclear	high	low	low	high
Luketich et al 2005	high	unclear	unclear	high	low	low	high
Bimston et al 1999	high	unclear	unclear	high	low	low	high

### Sensitivity analysis

We performed a sensitivity analysis of VAS scores at postoperative 4–8 and 24 h. We found that only when Bimston et al. [42] was excluded could heterogeneity be resolved at VAS 4–8 h, but the results did not change [MD 0.20; 95% CI:0.27 to 0.67; I^2^ = 46%; *p* = 0.05]. The exclusion of Bimston et al. [42] or Richardson et al. [35] resolved the heterogeneity of VAS scores at 24 h, but this also did not change the results. When we analyzed only data from studies with low risk of bias, we found no heterogeneity = 0%, but there was still no change in results.

### The primary outcomes: PVB versus EPI on the analgesic efficacy

**Figure 2:**
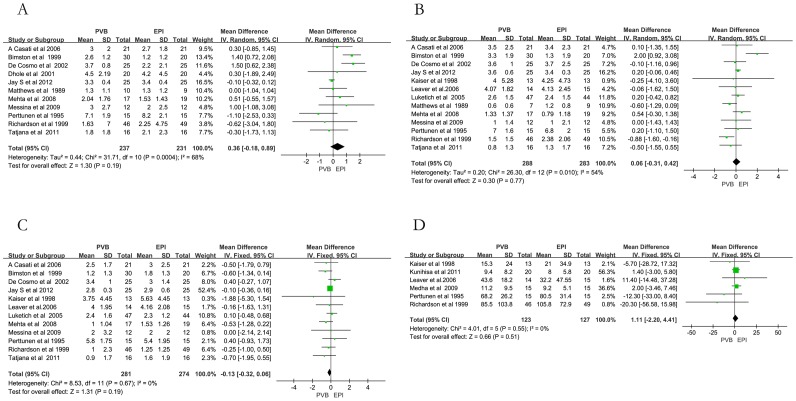
Meta-analyses of postoperative analgesic efficacy of PVB compared with that of EPI A) VAS scores 4–8 h; B) VAS scores 24 h; C) VAS scores 48 h; D) morphine consumption 24 h.

The trials assessed pain intensity using the VAS. There was no statistically significant difference in pain scores between the PVB and EPI groups at postoperative 4–8 h (MD 0.36; 95%CI: −0.18 to 0.89; I^2^ = 68%; *p* = 0.19; Fig. 2A), at 24 h (MD 0.06; 95%CI: −0.31 to 0.42; I^2^ = 54%; *p* = 0.77; Fig. 2B), or at 48 h (MD −0.13; 95%CI: −0.32 to 0.06; I^2^ = 0%; *p* = 0.19; Fig. 2C). There was also no significant difference in morphine consumption between the two groups at postoperative 24 h (MD 1.11; 95%CI: −2.20 to 4.41; I^2^ = 0%; *p* = 0.51; Fig. 2D).

### Comparison of adverse side effects

**Figure 3:**
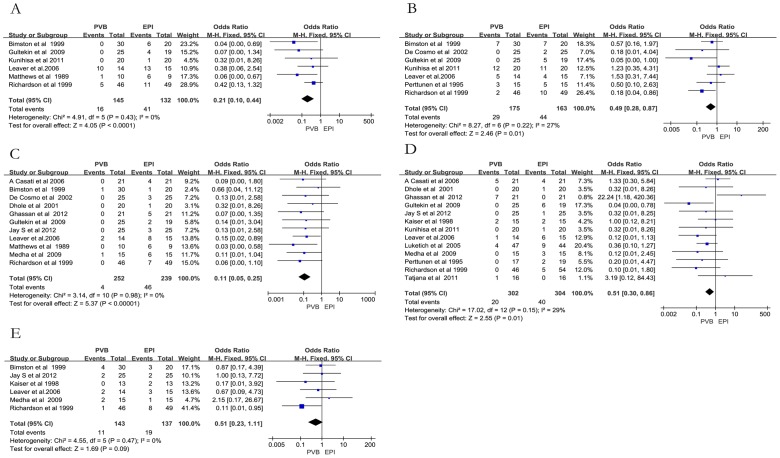
Meta-analyses of adverse side effect of PVB with that of EPI A) Urinary retention; B) nausea and vomiting; C) hypotension; D) rates of failed technique; E) pulmonary complications.

The analyzed adverse side effects consisted of pulmonary complication, urinary retention, nausea and vomiting, hypotension, and failed rates of technique (Table 3). Compared to EPI, PVB resulted in significantly less incidence rates of urinary retention (OR 0.21, 95%CI: 0.10 to 0.44; I^2^ = 0%; *p*<0.0001; Fig. 3A), nausea and vomiting (OR 0.49, 95% CI: 0.28 to 0.87; I^2^ = 27%, *p* = 0.01; Fig. 3B), and hypotension (OR 0.11, 95% CI: 0.05 to 0.25; I^2^ = 0%, *p*<0.00001; Fig. 3C). Rates of failed technique were lower in the PVB group (OR 0.51, 95%CI: 0.30 to 0.86; I^2^ = 29%; *p* = 0.01; Fig. 3D). However, there was no significant difference in pulmonary complications (OR 0.51, 95% CI: 0.23 to 1.11); I^2^ = 0%; *p* = 0.09; Fig. 3E).

**Table 3:** All detailed results.

		Heterogeneity		Test for overall effect		MD/OR	95% CI	Egger's Test	
		I^2^ (%)	P	Z	P			P	95% CI
Primary outcomes	VAS 4–8 h	68	0.0004	1.30	0.19	0.36	−0.18, 0.89	0.779	−4.81, 6.21
	VAS 24 h	54	0.01	0.30	0.77	0.06	−0.31, 0.42	0.923	−3.50, 3.83
	VAS 48 h	0	0.67	1.31	0.19	−0.13	−0.32, 0.06	0.218	−3.50, −0.90
	morphine usage 24 h	0	0.55	0.66	0.51	1.11	−2.20, 4.41	0.425	−2.88, 5.58
Secondary outcomes	Urinary retention	0	0.43	4.05	<0.0001	0.21	0.10, 0.44	0.007	−2.59, −0.77
	Nausea and Vomiting	27	0.22	2.46	0.01	0.49	0.28, 0.87	0.027	−3.50, −0.32
	Hypotension	0	0.98	5.37	<0.00001	0.11	0.05, 0.25	0.220	−1.91, 0.51
	Failed block	29	0.15	2.55	0.01	0.51	0.30, 0.86	0.448	−2.33, 1.18
	Pulmonary complications	0	0.47	1.69	0.09	0.51	0.23, 1.11	0.498	−6.11, 3.52

### Publication bias

Visual inspection of the funnel plot and Egger's test for publication bias (Figure S1, S2, S3, S4, S5, S6, S7, S8, S9, S10, S11, S12, S13, S14, S15, S16, S17, S18) suggests that there was no evidence of publication bias in VAS scores at postoperative 4–8 h (*p* = 0.779, 95% CI: −4.81 to 6.21), 24 h (*p* = 0.923, 95%CI: −3.5 to 3.83), 48 h (*p* = 0.218, 95% CI: −3.50 to 0.90), or for morphine usage (*p* = 0.425, 95% CI: −2.88 to 5.58), hypotension (*p* = 0.22, 95% CI: −1.91 to 0.51), rates of failed technique (*p* = 0.488, 95% CI: −2.33 to 1.18), or pulmonary complications (*p* = 0.498, 95% CI: −6.11 to 3.52). However, there was publication bias in urinary retention (*p* = 0.007, 95% CI: −2.59 to −0.77), and nausea and vomiting (*p* = 0.027, 95% CI: −3.5 to −0.32).

## Discussion

This updated meta-analysis, which included 777 patients, in 18 randomized controlled trials [10]–[18], [34]–[42] that compared PVB with EPI for thoracotomy, showed that PVB provides comparable analgesia with epidural blockade and furthermore has a better side effect profile. PVB is associated with less urinary retention, postoperative nausea and vomiting, and hypotension. These results were consistent with those of the meta-analysis performed by R. G. Davies in 2006 [9]. However, we also found that there were no significant differences between PVB and EPI in pulmonary complications. We assumed that the direct reason was the different concentration of an infusion of bupivacaine for PVB and EPI in Medha's study, the concentration was 0.25% and 0.125% respectively [17], resulted in the incidence of pneumonia was 1 patient (6.7%) in EPI, but 2 patients (13.3%) in PVB group. Bulger et al. [43] also demonstrated that epidural analgesia not only improved outcome for patients with chest wall pain but also decreased risk of nosocomial pneumonia. There was publication bias in urinary retention, nausea and vomiting, we think the reason is that studies with negative results were not published, in other words, positive results are easier to be reported.

Compared to the prior meta-analysis [9], approximately half of the articles included in the current study were new, and the quality of these studies was higher than before. Because of these characteristics, we consider this meta-analysis to be much more robust, and the result regarding pulmonary complications differs from the previous study.

Effective postoperative analgesic is believed to reduce morbidity, improve patient outcomes, and reduce hospital costs. Thoracic epidural analgesia is commonly used after thoracotomy. However, there are risks associated with the techniques such as neurological injury and paraplegia [44]. Sometimes, the epidural technique fails due to difficult anatomy [45].

Thoracic paravertebral block (PVB) is becoming increasingly popular in recent years. The classic technique described for PVB is a posterior approach using loss of resistance to air or saline as the superior costotransverse ligament is traversed [46]. Recent modifications to this technique have utilized ultrasound and nerve stimulation [47]. Alternatively, catheters can be placed in the paravertebral space intraoperatively under direct vision by the surgeon before chest closure [48]. These methods avoid some of the concerns regarding epidural placement in the presence of difficult anatomy, local sepsis, or impaired coagulation. More importantly, it can reduce the rate of neurological injury and paraplegia.

Many studies have shown thoracic PVB to be an effective form of analgesia after thoracotomy, multiple fractured ribs, major breast surgery, and inguinal hernia repair [49]. Andreae et al. [6] concluded that Paravertebral block reduced the risk of chronic pain after breast cancer surgery in about one of every 5 women. Schnabel et al. [50] in 2010 also reported that perioperative PVB is a feasible and effective method for improved postoperative pain after breast surgery. Thavaneswaran et al. [51] concluded that PVB can be applied during herniorrhaphy. Although our meta-analysis showed that there was no difference in pain scores and pulmonary complications between PVB and EPI, there was a statistically significant improvement in PVB in terms of adverse side effects.

### Limitations

This meta-analysis is characterized by several limitations that should be noted. Firstly, the findings are based on relatively low quality data with a high risk of bias. This is a common limitation of systematic reviews. In addition, only papers written in English were included. Secondly, surgical placement of the catheter under direct vision must influence the results of side effects because it avoids complications and reduces failure rates. Thirdly, various drug regimens were implemented for EPI and PVB. In contrast to the studies of Richardson et al. [35] and Casati et al. [12], in which only a local anesthetic solution was used, Tatjana et al. [16] administrated an infusion of a local anesthetic-opioid combination to both group. This influences not only analgesic efficacy but also respiratory depression, because a combination of local anesthetic and opioid administration carries a high risk of respiratory depression.

## Conclusions

Our analysis represents a least-biased attempt to pool the results of several studies. A large, prospective, randomized trial is necessary to confirm these findings. Extensive, large, randomized, double-blind, multicenter, controlled clinical trials that compared PVB and EPI will be better.

This meta-analysis showed that PVB can provide comparable pain relief to traditional EPI, and may have a better side-effect profile for pain relief after thoracic surgery. Further high-powered randomized trials are to need to determine whether PVB truly offers any advantages over EPI.

## Supporting Information

Figure S1 **Egger's test for primary and secondary outcomes.**
(TIF)

Figure S2 **Egger's test for primary and secondary outcomes.**
(TIF)

Figure S3 **Egger's test for primary and secondary outcomes.**
(TIF)

Figure S4 **Egger's test for primary and secondary outcomes.**
(TIF)

Figure S5 **Egger's test for primary and secondary outcomes.**
(TIF)

Figure S6 **Egger's test for primary and secondary outcomes.**
(TIF)

Figure S7 **Egger's test for primary and secondary outcomes.**
(TIF)

Figure S8 **Egger's test for primary and secondary outcomes.**
(TIF)

Figure S9 **Egger's test for primary and secondary outcomes.**
(TIF)

Figure S10 **Funnel plot for primary and secondary outcomes.**
(TIF)

Figure S11 **Funnel plot for primary and secondary outcomes.**
(TIF)

Figure S12 **Funnel plot for primary and secondary outcomes.**
(TIF)

Figure S13 **Funnel plot for primary and secondary outcomes.**
(TIF)

Figure S14 **Funnel plot for primary and secondary outcomes.**
(TIF)

Figure S15 **Funnel plot for primary and secondary outcomes.**
(TIF)

Figure S16 **Funnel plot for primary and secondary outcomes.**
(TIF)

Figure S17 **Funnel plot for primary and secondary outcomes.**
(TIF)

Figure S18 **Funnel plot for primary and secondary outcomes.**
(TIF)

Checklist S1 **PRISMA 2009 Checklist.**
(DOC)

Appendix S1 **The Cochrane Library search strategy.**
(DOC)

## References

[1] Kavanagh BP, Katz J, Sandler AN (1994) Pain control after thoracic surgery. A review of current techniques. Anesthesiology 81: 737–59. 8092520 10.1097/00000542-199409000-00028

[2] Sabanathan S, Eng J, Mearns AJ (1990) Alterations in respiratory mechanics following thoracotomy. J R Coll Surg Edinb 35: 144–50. 2203902

[3] Plumis WA, Steegers MA, Verhagen AF, Scheffer GJ, Wilder-Smith OH (2006) Chronic post-thoracotomy pain: A retrospective study. Acta Anaesthesiol Scand 50: 804–808. 16879462 10.1111/j.1399-6576.2006.01065.x

[4] Soto RG, Fu ES (2003) Acute pain management for patients undergoing thoracotomy. Ann Thorc Surg 75: 1349–1357. 10.1016/s0003-4975(02)04647-7 12683601

[5] Joshi GP, Bonnet F, Shah R, Wilkinson RC, Camu F, et al (2008) A systematic review of randomized trials evaluating regional techniques for postthoracotomy analgesia. Anesth Analg 107(3): 1026–40. 18713924 10.1213/01.ane.0000333274.63501.ff

[6] Andreae MH, Andreae DA (2012) Local anaesthetics and regional anaesthesia for preventing chronic pain after surgery. Cochrane Database Syst Rev 17: ; 10. 10.1002/14651858.CD007105.pub2 PMC4004344 23076930

[7] Hansdottir V, Philip J, Olsen MF, Eduard C, Houltz E, et al (2006) Thoracic epidural versus intravenous patient-controlled analgesia after cardiac surgery. Anesthesiology 104: 142–151. 16394700 10.1097/00000542-200601000-00020

[8] Horlocker TT (2003) Thromboprophylaxis and neuraxial anesthesia. Orthopedics 26: S243–S249. 12597233 10.3928/0147-7447-20030202-08

[9] Davis RG, Myles PS, Graham JM (2006) A comparison of the analgesic efficacy and side effects of paravertebral vs. epidural blockade for thoracotomy—A systematic review and meta-analysis of randomized trial. Br J Anaesth 96: 418–426. 16476698 10.1093/bja/ael020

[10] Hotta Kunihisa, Endo T, Taira K, Sata N, Inoue S, et al (2011) Comparison of the analgesic effects of continuous extrapleural block and continuous epidural block after video-assisted thoracoscopic surgery. J Cardiothorac Vasc Anesth 25: 1009–13. 21955830 10.1053/j.jvca.2011.07.026

[11] Grider JS, Mullet TW, Saha SP, Harned ME, Sloan PA (2012) A randomized, double-blind trial comparing continuous thoracic epidural bupivacaine with and without opioid in contrast to a continuous paravertebral infusion of bupivacaine for post-thoracotomy pain. J Cardiothorac Vasc Anesth 26: 83–9. 22100213 10.1053/j.jvca.2011.09.003

[12] Casati A, Alessandrini P, Nuzzi M, Tosi M, Iotti E, et al (2006) A prospective, randomized, blinded comparison between continuous thoracic paravertebral and epidural infusion of 0.2% ropivacaine after lung resection surgery. Eur J Anaesthesiol 23: 999–1004. 16824243 10.1017/S0265021506001104

[13] Mehta Y, Arora D, Sharma KK, Mishra Y, Wasir H, et al (2008) Comparison of continuous thoracic epidural and paravertebral block for postoperative analgesia after robotic-assisted coronary artery bypass surgery. Ann Card Anaesth 11: 91–6. 18603748 10.4103/0971-9784.41576

[14] Gulbahar G, Kocer B, Muratli SN, Yildirim E, Gulbahar O, et al (2010) A comparison of epidural and paravertebral catheterisation techniques in post-thoracotomy pain management. Eur J Cardiothorac Surg 37: 467–72. 19709893 10.1016/j.ejcts.2009.05.057

[15] Messina M, Boroli F, Landoni G, Bignami E, Dedola E, et al (2009) A comparison of epidural vs. paravertebral blockade in thoracic surgery. Minerva Anestesiol 75: 616–21. 19881458

[16] Pintaric TS, Potocnik I, Hadzic A, Stupnik T, Pintaric M, et al (2011) Comparison of continuous thoracic epidural with paravertebral block on perioperative analgesia and hemodynamic stability in patients having open lung surgery. Reg Anesth Pain Med 36: 256–60. 21490523 10.1097/AAP.0b013e3182176f42

[17] Mohta M, Verma P, Saxena AK, Sethi AK, Tyagi A, et al (2009) Prospective, randomized comparison of continuous thoracic epidural and thoracic paravertebral infusion in patients with unilateral multiple fractured ribs–a pilot study. J Trauma 66: 1096–101. 19359920 10.1097/TA.0b013e318166d76d

[18] Kanazi GE, Ayoub CM, Aouad M, Abdallah F, Sfeir PM, et al (2012) Subpleural block is less effective than thoracic epidural analgesia for post-thoracotomy pain: a randomised controlled study. Eur J Anaesthesiol 29: 186–91. 22327109 10.1097/EJA.0b013e32834fcef7

[19] Higgins JPT, Altman DG (2008) Assessing risk of bias in included studies. In: Higgins JPT, Green S, eds. Cochrane handbook for systematic reviews of interventions Wiley 187–241.

[20] Egger M, Smith GD, Phillips AN (1997) Meta-analysis: principles and procedures. Br Med J 315: 1533–7. 9432252 10.1136/bmj.315.7121.1533 PMC2127925

[21] Kaya FN, Turker G, Mogol EB, Bayraktar S (2012) Thoracic paravertebral block for video-assisted thoracoscopic surgery: single injection versus multiple injections. J Cardiothorac Vasc Anesth 26: 90–4. 22055006 10.1053/j.jvca.2011.09.008

[22] Helms O, Mariano J, Hentz JG, Santelmo N, Falcoz PE, et al (2011) Intra-operative paravertebral block for postoperative analgesia in thoracotomy patients: a randomized, double-blind, placebo-controlled study. Eur J Cardiothorac Surg 40: 902–6. 21377888 10.1016/j.ejcts.2011.01.067

[23] Liang Y, Chu H, Zhen H, Wang S, Gu M (2012) A prospective randomized study of intraoperative thoracic epidural analgesia in off-pump coronary artery bypass surgery. J Anesth 26: 393–9. 22274169 10.1007/s00540-012-1325-6

[24] Fortier S, Hanna HA, Bernard A, Girard C (2012) Comparison between systemic analgesia, continuous wound catheter analgesia and continuous thoracic paravertebral block: a randomised, controlled trial of postthoracotomy pain management. Eur J Anaesthesiol 29: 524–30. 22914044 10.1097/EJA.0b013e328357e5a1

[25] Esme H, Apiliogullari B, Duran FM, Yoldas B, Bekci TT (2012) Comparison between intermittent intravenous analgesia and intermittent paravertebral subpleural analgesia for pain relief after thoracotomy. Eur J Cardiothorac Surg 41: 10–3. 21596578 10.1016/j.ejcts.2011.03.056 PMC3241091

[26] Hill SE, Keller RA, Stafford-Smith M, Grichnik K, White WD, et al (2006) Efficacy of single-dose, multilevel paravertebral nerve blockade for analgesia after thoracoscopic procedures. Anesthesiology 104: 1047–53. 16645458 10.1097/00000542-200605000-00022

[27] Yazigi A, Abou-Zeid H, Srouji T, Madi-Jebara S, Haddad F, et al (2012) The effect of low-dose intravenous ketamine on continuous intercostal analgesia following thoracotomy. Ann Card Anaesth 15: 32–8. 22234019 10.4103/0971-9784.91479

[28] Mustola ST, Lempinen J, Saimanen E, Vilkko P (2011) Efficacy of thoracic epidural analgesia with or without intercostal nerve cryoanalgesia for postthoracotomy pain. Ann Thorac Surg 91: 869–73. 21353017 10.1016/j.athoracsur.2010.11.014

[29] Fibla JJ, Molins L, Mier JM, Sierra A, Vidal G (2009) A prospective study of analgesic quality after a thoracotomy: paravertebral block with ropivacaine before and after rib spreading. Eur J Cardiothorac Surg 36: 901–5. 19615915 10.1016/j.ejcts.2009.05.041

[30] Garutti I, González-Aragoneses F, Biencinto MT, Novoa E, Simón C, et al (2009) Thoracic paravertebral block after thoracotomy: comparison of three different approaches. Eur J Cardiothorac Surg 35: 829–32. 19318275 10.1016/j.ejcts.2009.01.025

[31] Ju H, Feng Y, Yang BX, Wang J (2008) Comparison of epidural analgesia and intercostal nerve cryoanalgesia for post-thoracotomy pain control. Eur J Pain 12: 378–84. 17870625 10.1016/j.ejpain.2007.07.011

[32] Hura G, Knapik P, Misiołek H, Krakus A, Karpe J (2006) Sensory blockade after thoracic paravertebral injection of ropivacaine or bupivacaine. Eur J Anaesthesiol 23: 658–64. 16805930 10.1017/S0265021506000561

[33] Ryu HG, Lee CJ, Kim YT, Bahk JH (2011) Preemptive low-dose epidural ketamine for preventing chronic postthoracotomy pain: a prospective, double-blinded, randomized, clinical trial. Clin J Pain 27: 304–8. 21178605 10.1097/AJP.0b013e3181fd5187

[34] Kaiser AM, Zollinger A, De Lorenzi D, Largiadèr F, Weder W (1998) Prospective, randomized comparison of extrapleural versus epidural analgesia for postthoracotomy pain. Ann Thorac Surg 66: 367–72. 9725371 10.1016/s0003-4975(98)00448-2

[35] Richardson J, Sabanathan S, Jones J, Shah RD, Cheema S, et al (1999) A prospective, randomized comparison of preoperative and continuous balanced epidural or paravertebral bupivacaine on post-thoracotomy pain, pulmonary function and stress responses. Br J Anaesth 83: 387–92. 10655907 10.1093/bja/83.3.387

[36] Leaver A, Yeomans M, Shelton A (2006) A randomized trial comparing thoracic epidural with paravertebral blocks for postoperative analgesia after pneumonectomy.

[37] Matthews PJ, Govenden V (1989) Comparison of continuous paravertebral and extradural infusions of bupivacaine for pain relief after thoracotomy. Br J Anaesth 62: 204–5. 2923769 10.1093/bja/62.2.204

[38] De Cosmo G, Aceto P, Campanale E (2002) Comparison between epidural and paravertebral intercostal nerve block with ropivacaine after thoracotomy: Effects on pain relief, pulmonary function and patient satisfaction. Acta Med Rom 40: 340–7.

[39] Perttunen K, Nilsson E, Heinonen J, Hirvisalo EL, Salo JA, et al (1995) Extradural, paravertebral and intercostal nerve blocks for post-thoracotomy pain. Br J Anaesth 75: 541–7. 7577277 10.1093/bja/75.5.541

[40] Dhole S, Mehta Y, Saxena H, Juneja R, Trehan N (2001) Comparison of continuous thoracic epidural and paravertebral blocks for post-operative analgesia after minimally invasive direct coronary artery bypass surgery. J Cardiothorac Vasc Anesth 15: 288–92. 11426357 10.1053/jcan.2001.23271

[41] Luketich JD, Land SR, Sullivan EA, Alvelo-Rivera M, Ward J, et al (2005) Thoracic epidural versus intercostal nerve catheter plus patient-controlled analgesia: a randomized study. Ann Thorac Sur 79: 1845–9. 10.1016/j.athoracsur.2004.10.055 15919269

[42] Bimston DN, McGee JP, Liptay MJ, Fry WA (1999) Continuous Paravertebral extrapleural infusion for post-thoracotomy pain management. Surgery 126: 650–6. 10520911

[43] Bulger EM, Edwards T, Klotz P, Jurkovich GJ (2004) Epidural analgesia improves outcome after multiple rib fractures. Surgery 136: 426–430. 15300210 10.1016/j.surg.2004.05.019

[44] Grant RP (1999) Con: every postthoracotomy patient does not deserve thoracic epidural analgesia. J Cardiothorac Vasc Anesth 13: 355–7. 10392691 10.1016/s1053-0770(99)90277-x

[45] Arendt K, Segal S (2008) Why epidural do not always work. Rev Obstet Gynecol 1: 49–55. 18769661 PMC2505163

[46] Eason MJ, Wyatt R (1979) Paravertebral thoracic block—a reappraisal. Anaesthesia 34: 638–42. 517716 10.1111/j.1365-2044.1979.tb06363.x

[47] Lang SA (2002) The use of a nerve stimulator for thoracic paravertebral block. Anesthesiology 97: 521. 12151950 10.1097/00000542-200208000-00037

[48] Sabanathan S, Smith PJ, Pradhan GN, Hashimi H, Eng JB, et al (1988) Continuous intercostal nerve block for pain relief after thoracotomy. Ann Thorac Surg 46: 425–6. 3178353 10.1016/s0003-4975(10)64657-7

[49] Karmakar MK (2001) Thoracic paravertebral block. Anesthesiology 95: 771–80. 11575553 10.1097/00000542-200109000-00033

[50] Schnabel A, Reichl SU, Kranke P, Pogatzki-Zahn EM, Zahn PK (2010) Efficacy and safety of paravertebral blocks in breast surgery: a meta-analysis of randomized controlled trials. Br J Anaesth 105: 842–52. 20947592 10.1093/bja/aeq265

[51] Thavaneswaran P, Rudkin GE, Cooter RD, Moyes DG, Perera CL, et al (2010) Brief reports: paravertebral block for anesthesia: a systematic review. Anesth Analg 110: 1740–4. 20448076 10.1213/ANE.0b013e3181da82c8

