# Supplementary figures and images for: A Comparison of the Analgesia Efficacy and Side Effects of Paravertebral Compared with Epidural Blockade for Thoracotomy: An Updated Meta-Analysis
Source: PLoS One. 2014 May 5;9(5):e96233. doi: 10.1371/journal.pone.0096233 (PMC4010440; doi:10.1371/journal.pone.0096233)

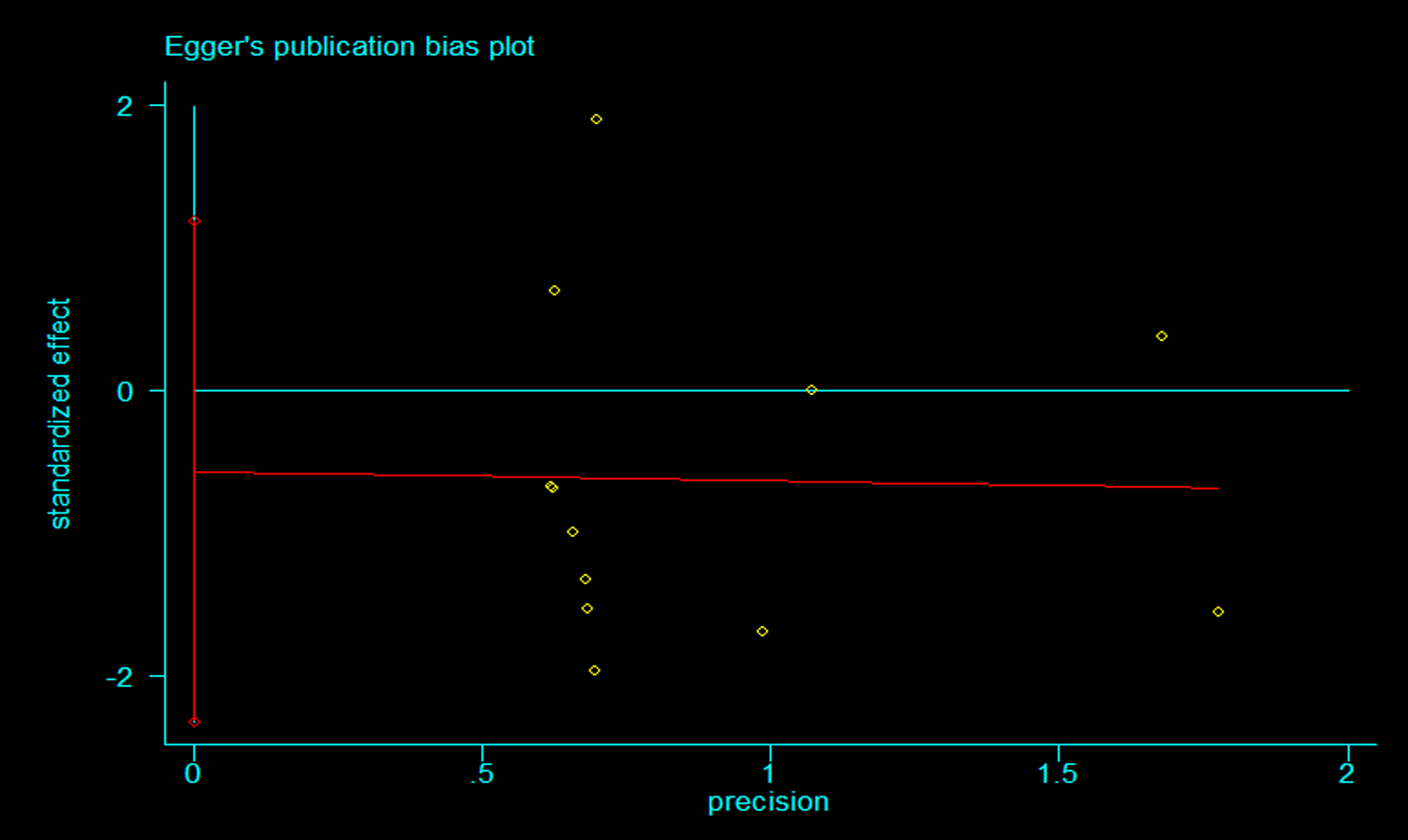

Supplement: Figure S1 — Egger's test for primary and secondary outcomes. (TIF) [file pone.0096233.s001.tif]

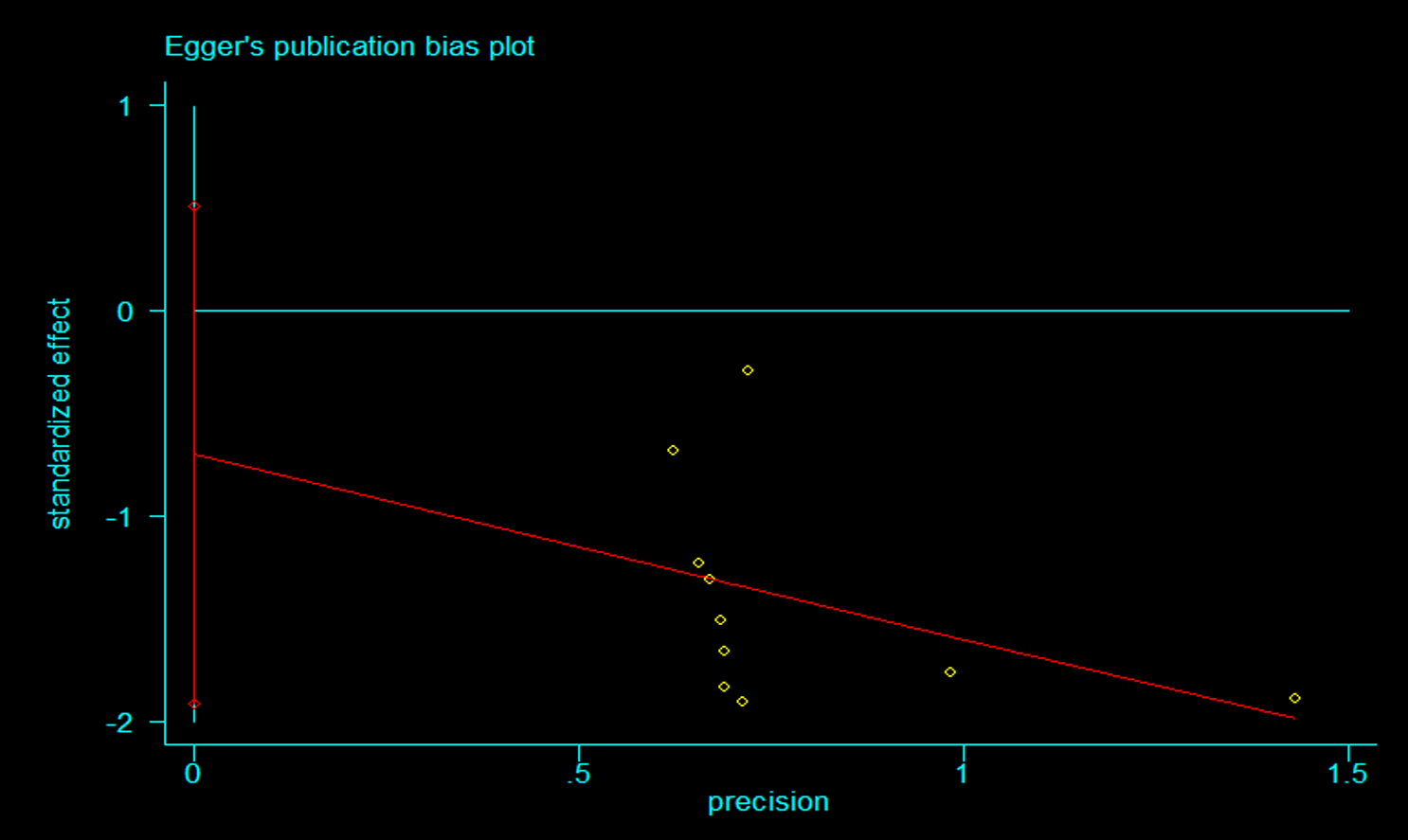

Supplement: Figure S2 — Egger's test for primary and secondary outcomes. (TIF) [file pone.0096233.s002.tif]

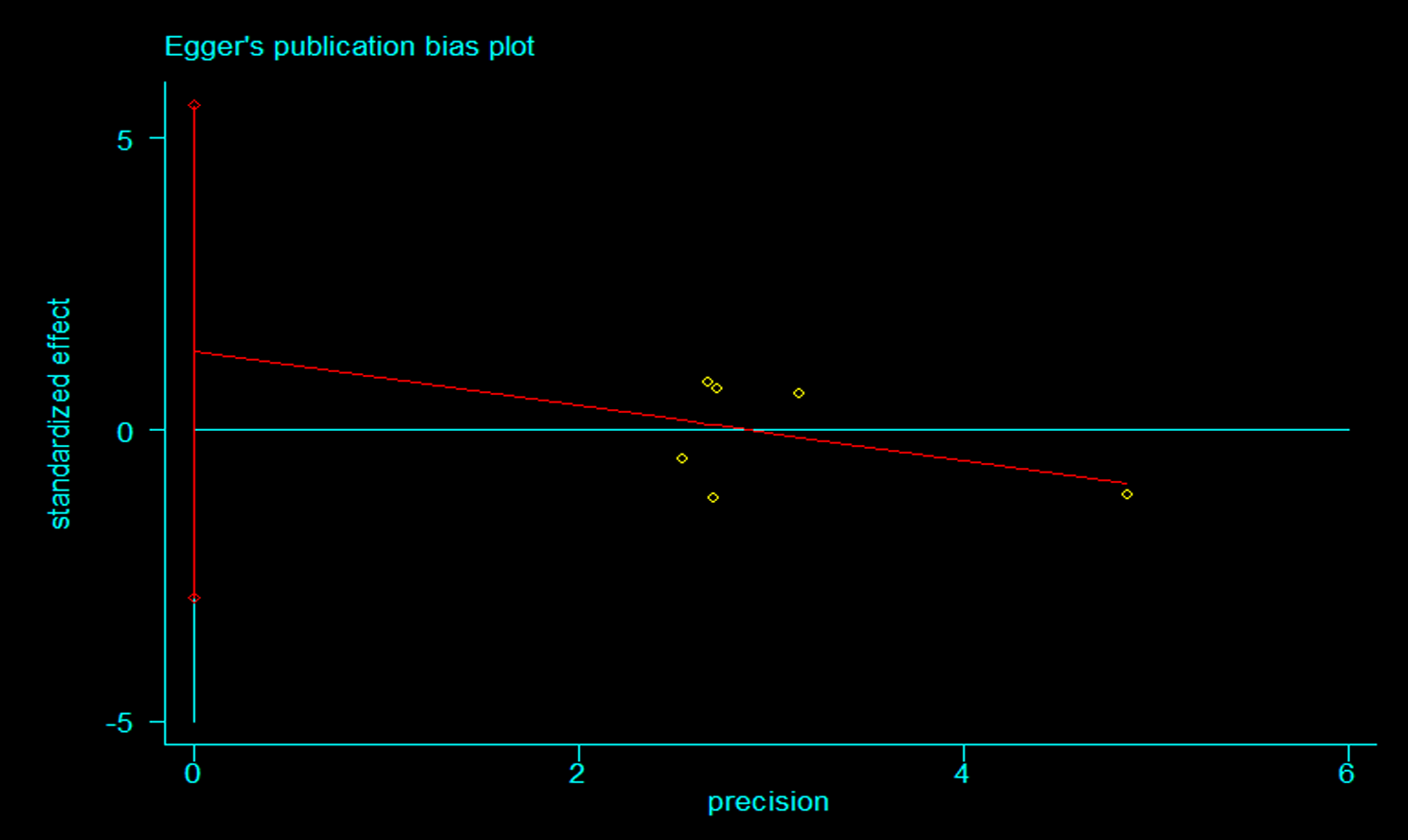

Supplement: Figure S3 — Egger's test for primary and secondary outcomes. (TIF) [file pone.0096233.s003.tif]

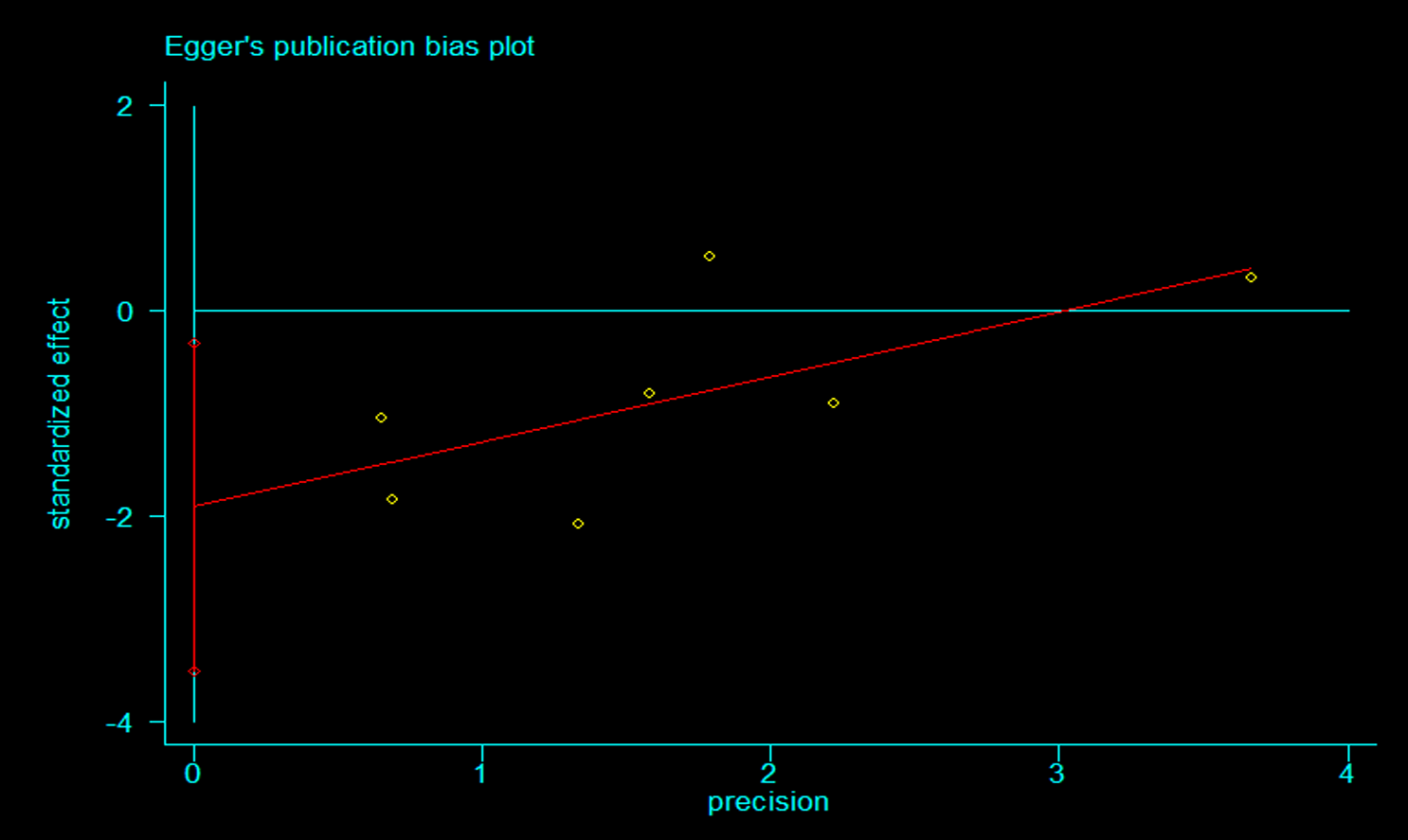

Supplement: Figure S4 — Egger's test for primary and secondary outcomes. (TIF) [file pone.0096233.s004.tif]

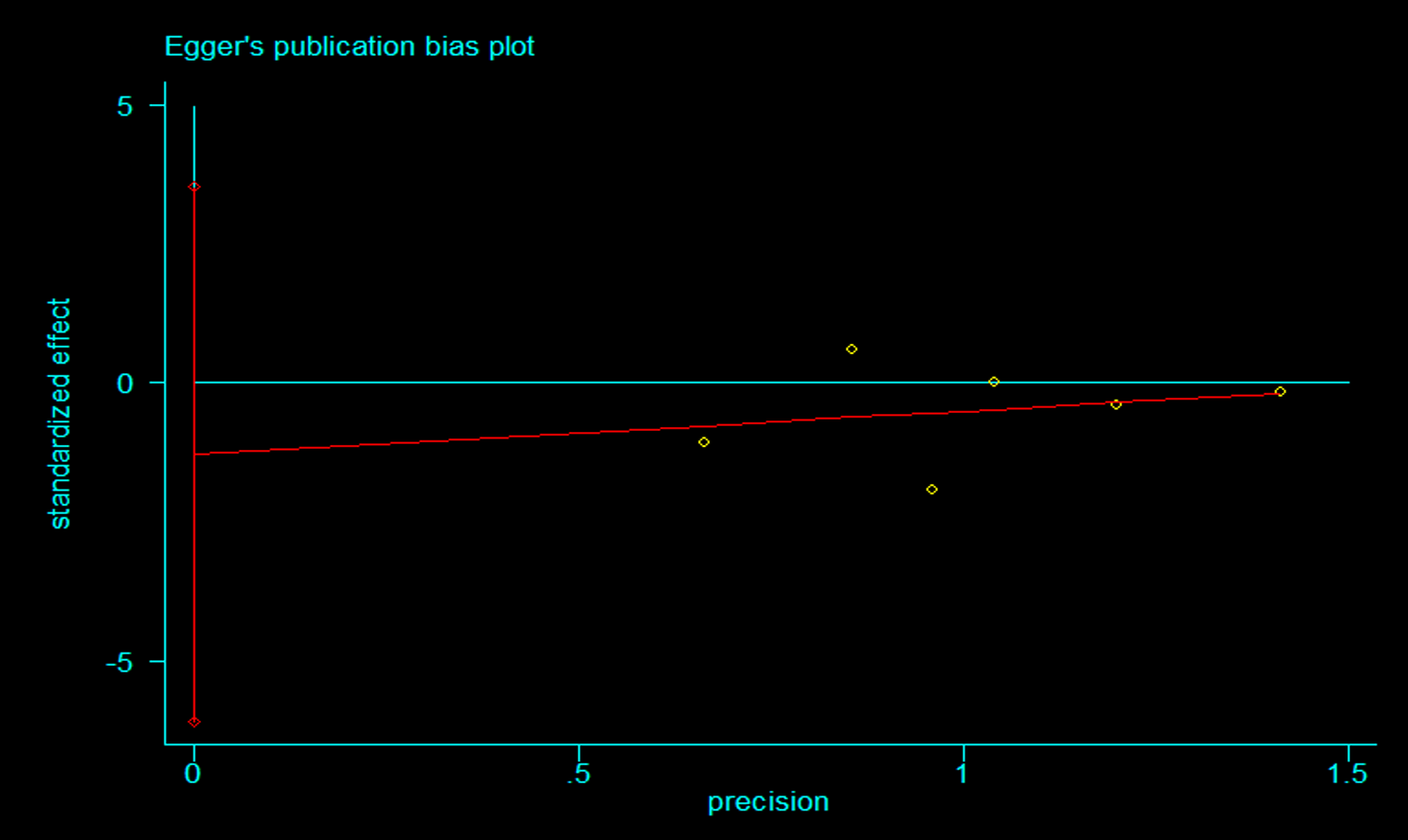

Supplement: Figure S5 — Egger's test for primary and secondary outcomes. (TIF) [file pone.0096233.s005.tif]

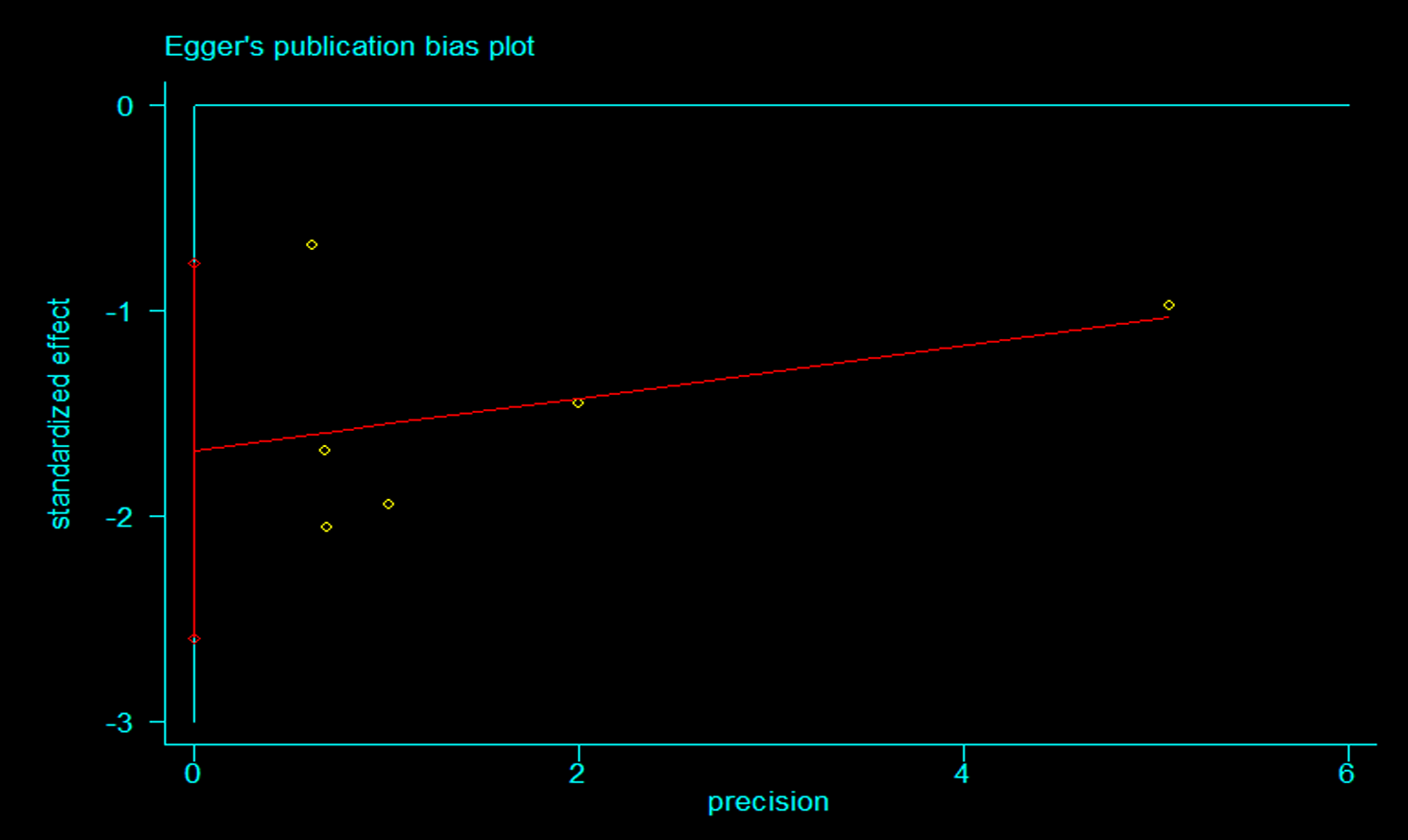

Supplement: Figure S6 — Egger's test for primary and secondary outcomes. (TIF) [file pone.0096233.s006.tif]

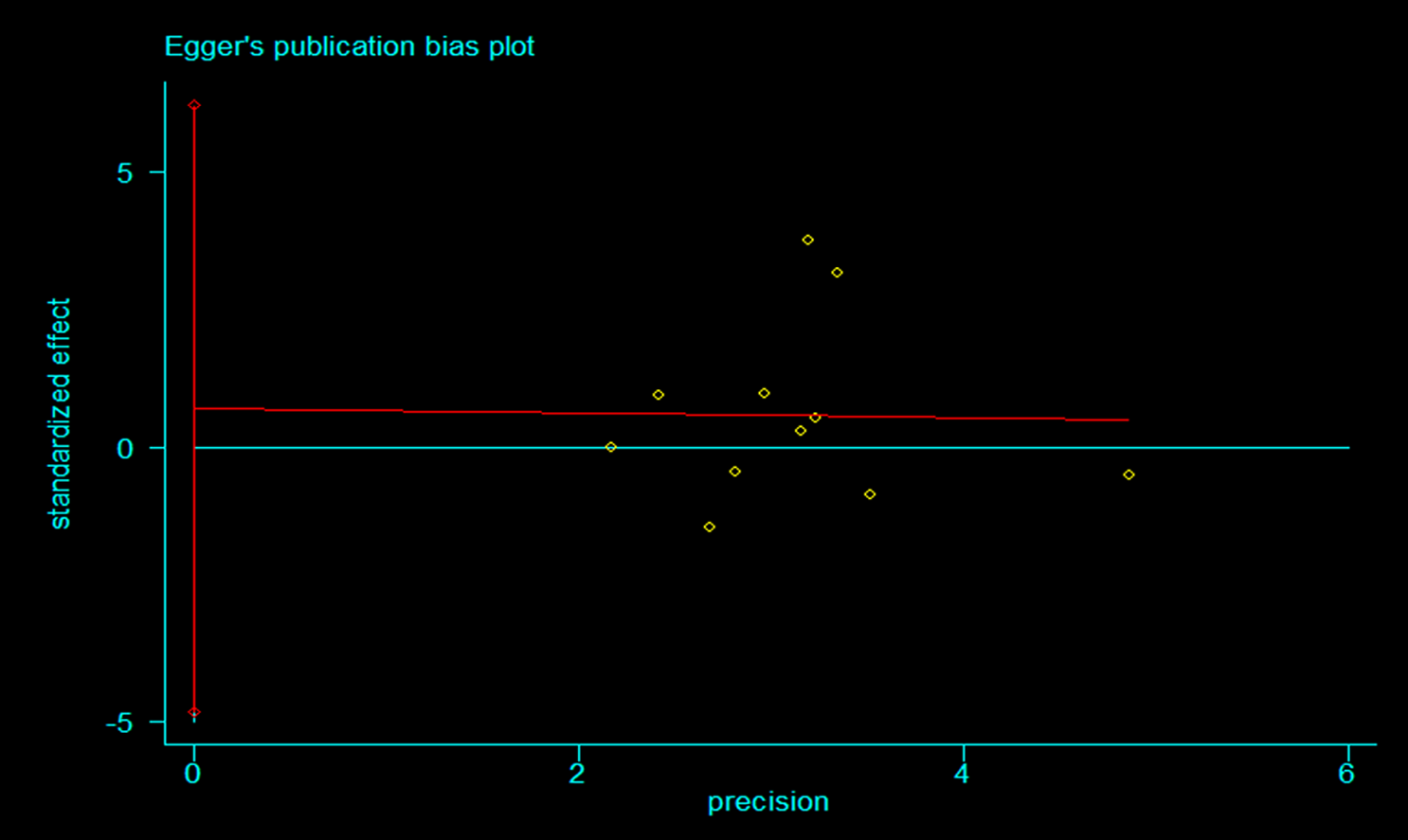

Supplement: Figure S7 — Egger's test for primary and secondary outcomes. (TIF) [file pone.0096233.s007.tif]

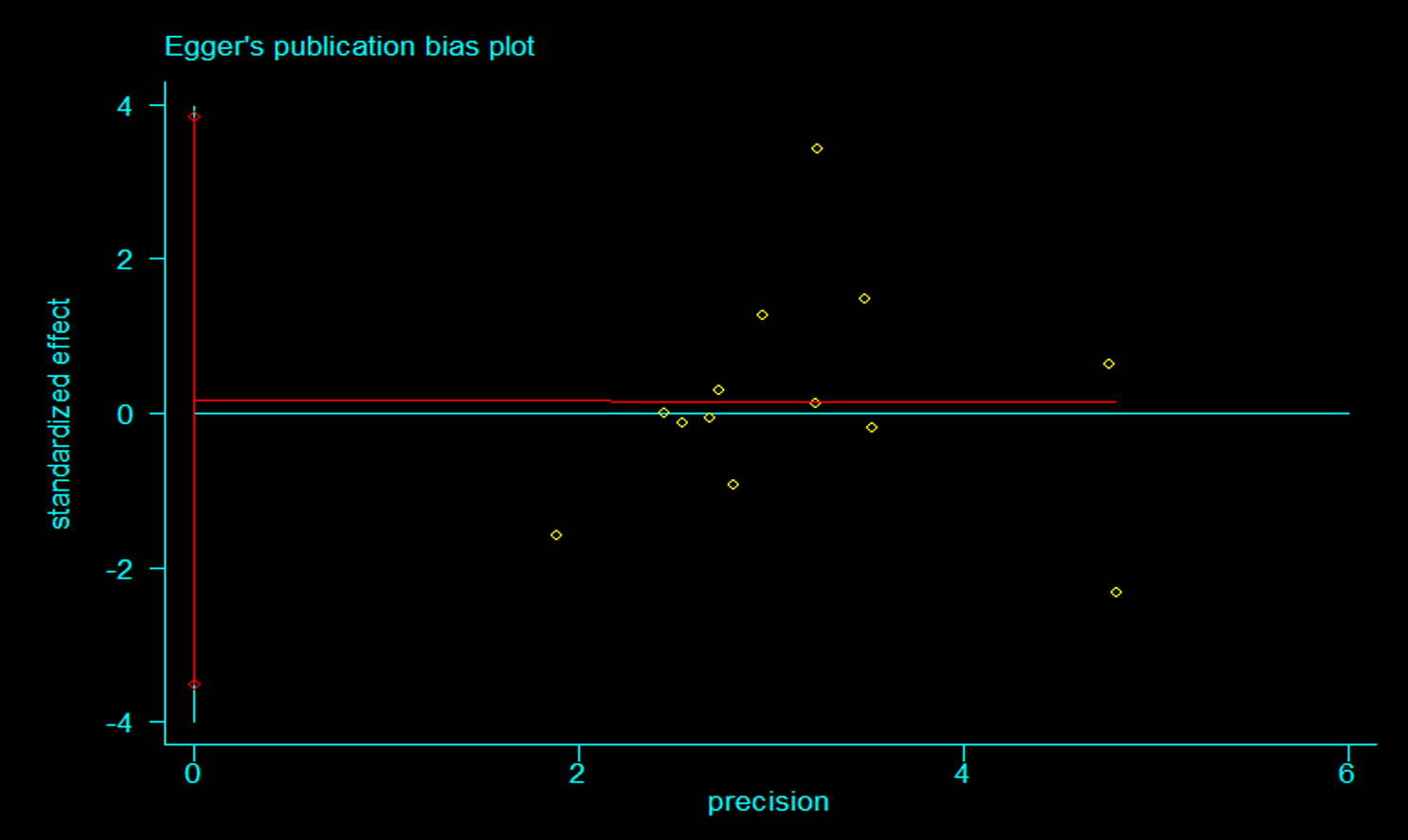

Supplement: Figure S8 — Egger's test for primary and secondary outcomes. (TIF) [file pone.0096233.s008.tif]

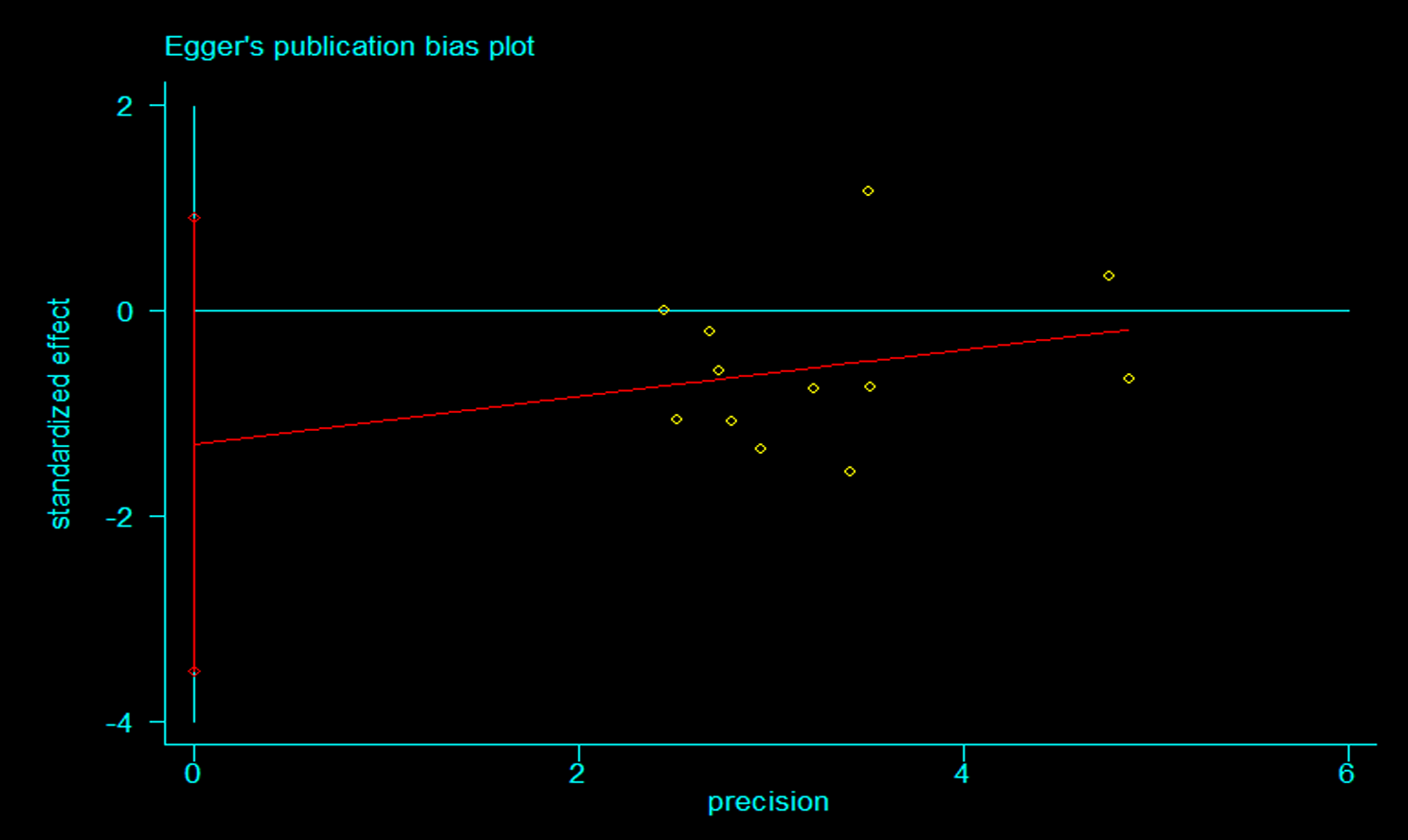

Supplement: Figure S9 — Egger's test for primary and secondary outcomes. (TIF) [file pone.0096233.s009.tif]

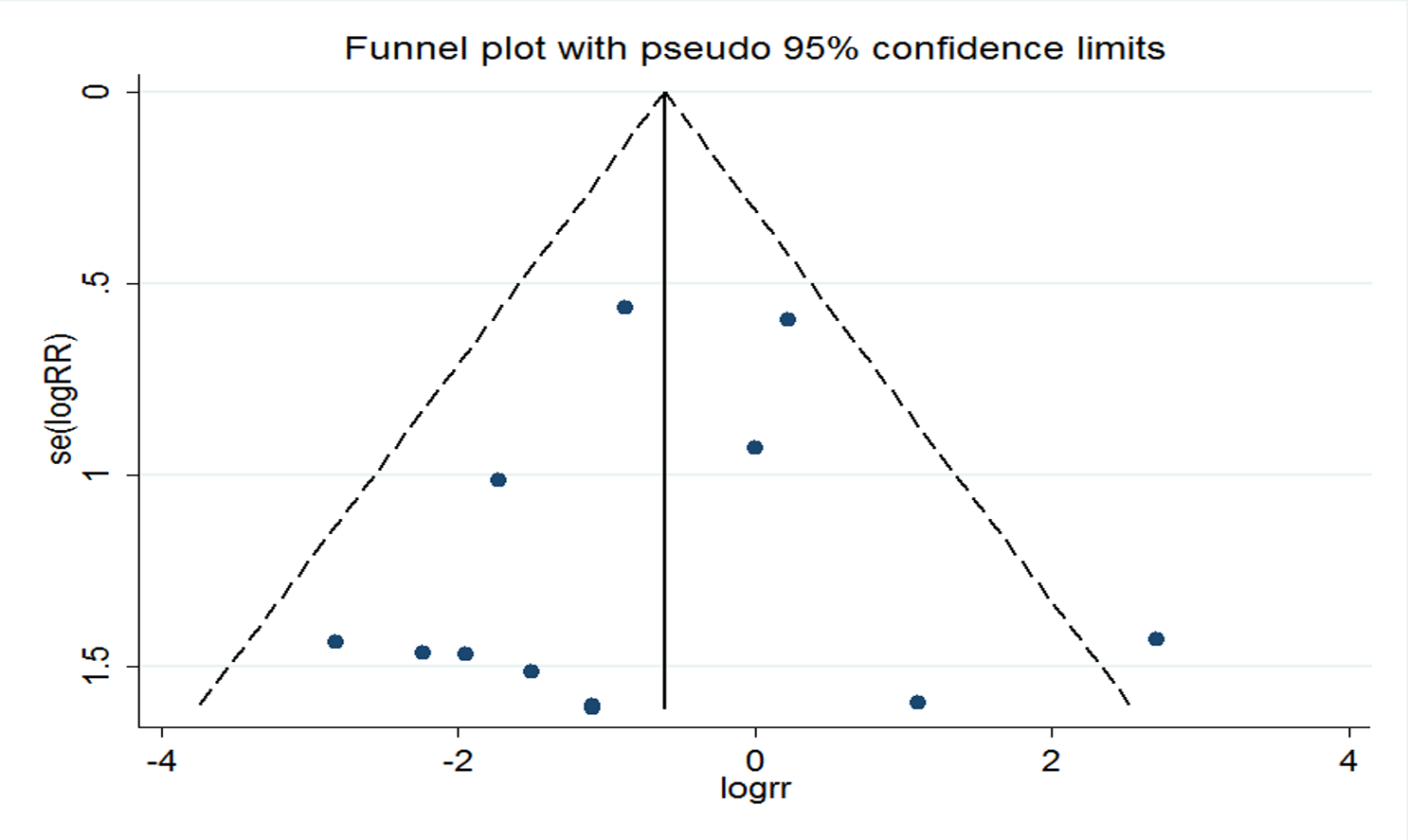

Supplement: Figure S10 — Funnel plot for primary and secondary outcomes. (TIF) [file pone.0096233.s010.tif]

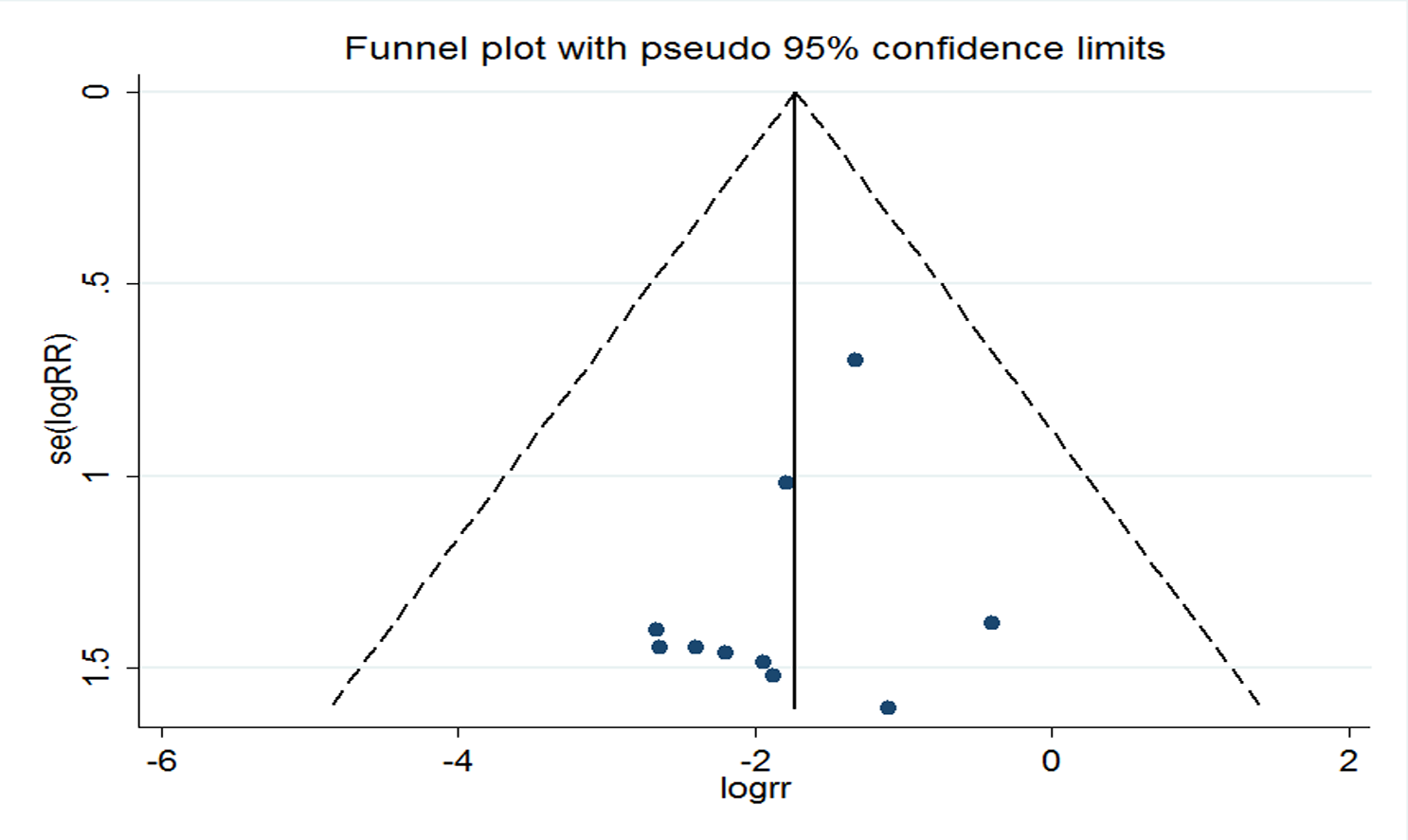

Supplement: Figure S11 — Funnel plot for primary and secondary outcomes. (TIF) [file pone.0096233.s011.tif]

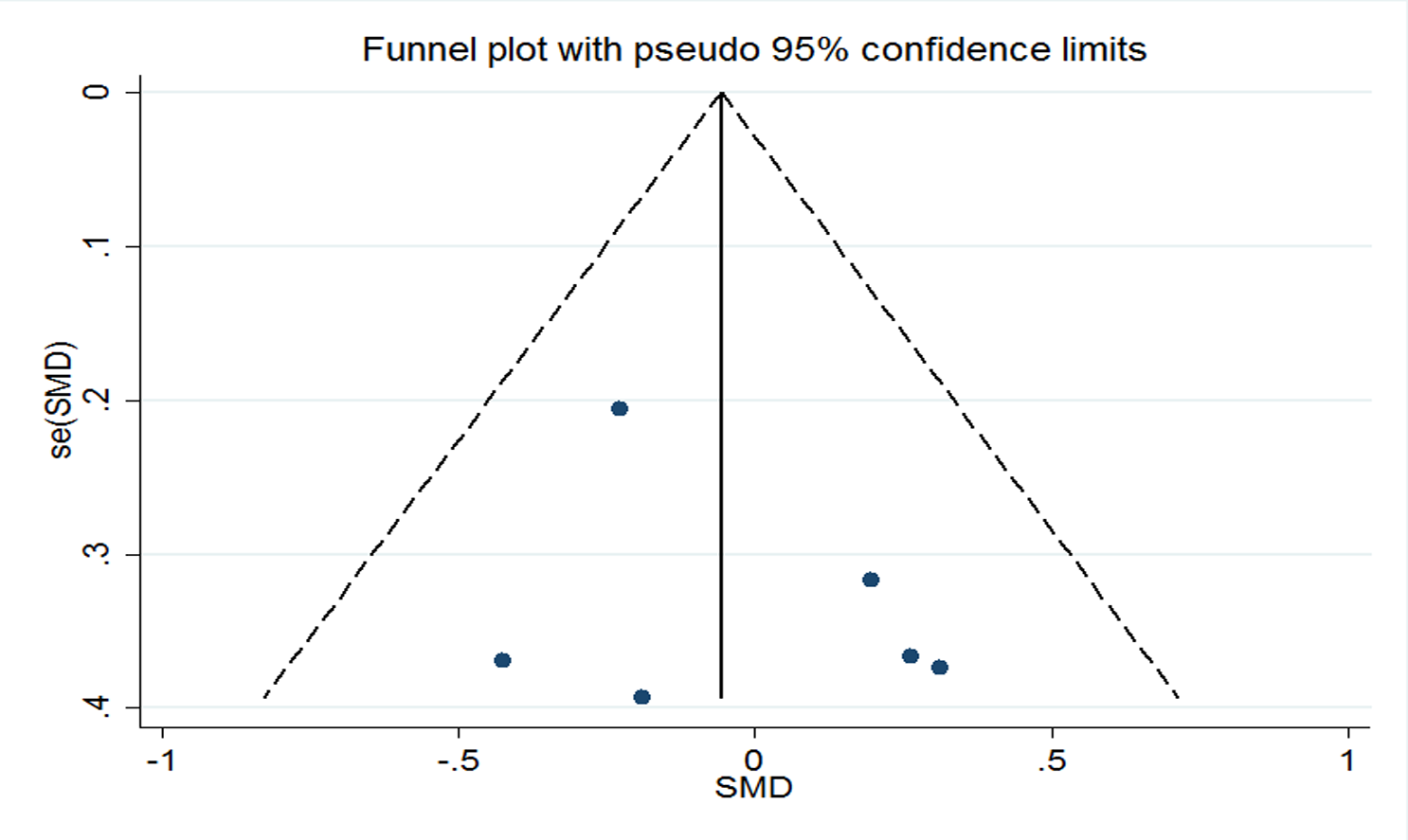

Supplement: Figure S12 — Funnel plot for primary and secondary outcomes. (TIF) [file pone.0096233.s012.tif]

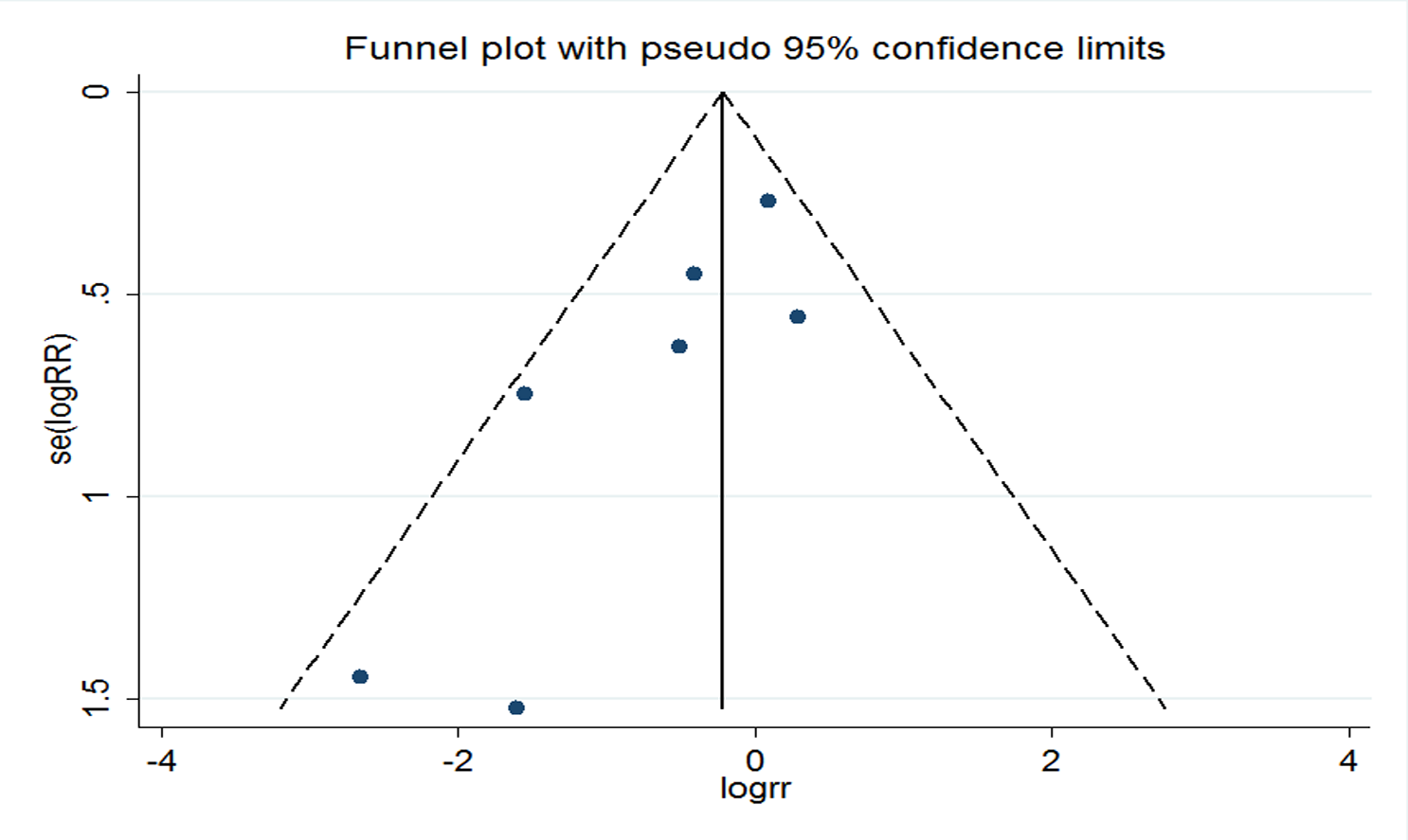

Supplement: Figure S13 — Funnel plot for primary and secondary outcomes. (TIF) [file pone.0096233.s013.tif]

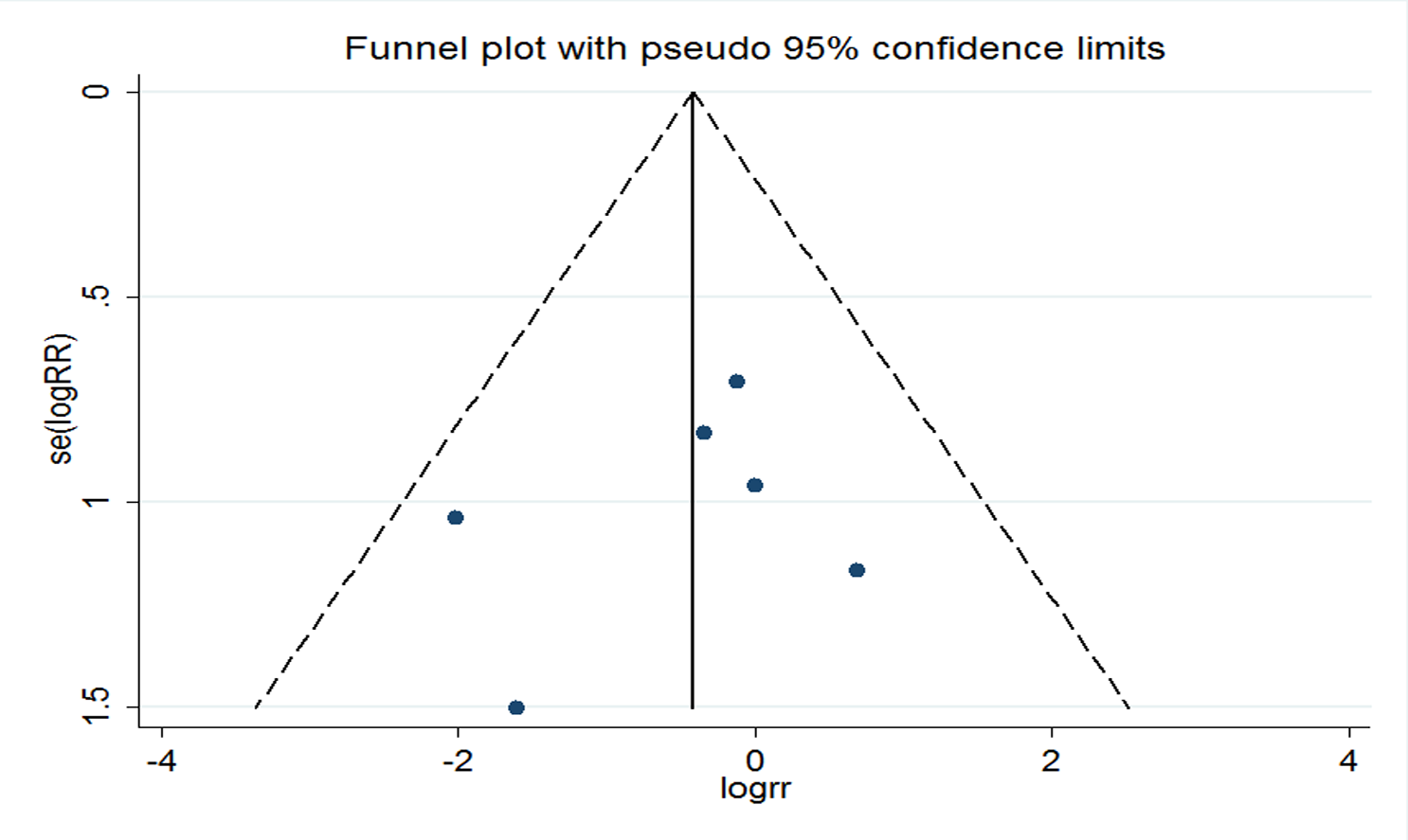

Supplement: Figure S14 — Funnel plot for primary and secondary outcomes. (TIF) [file pone.0096233.s014.tif]

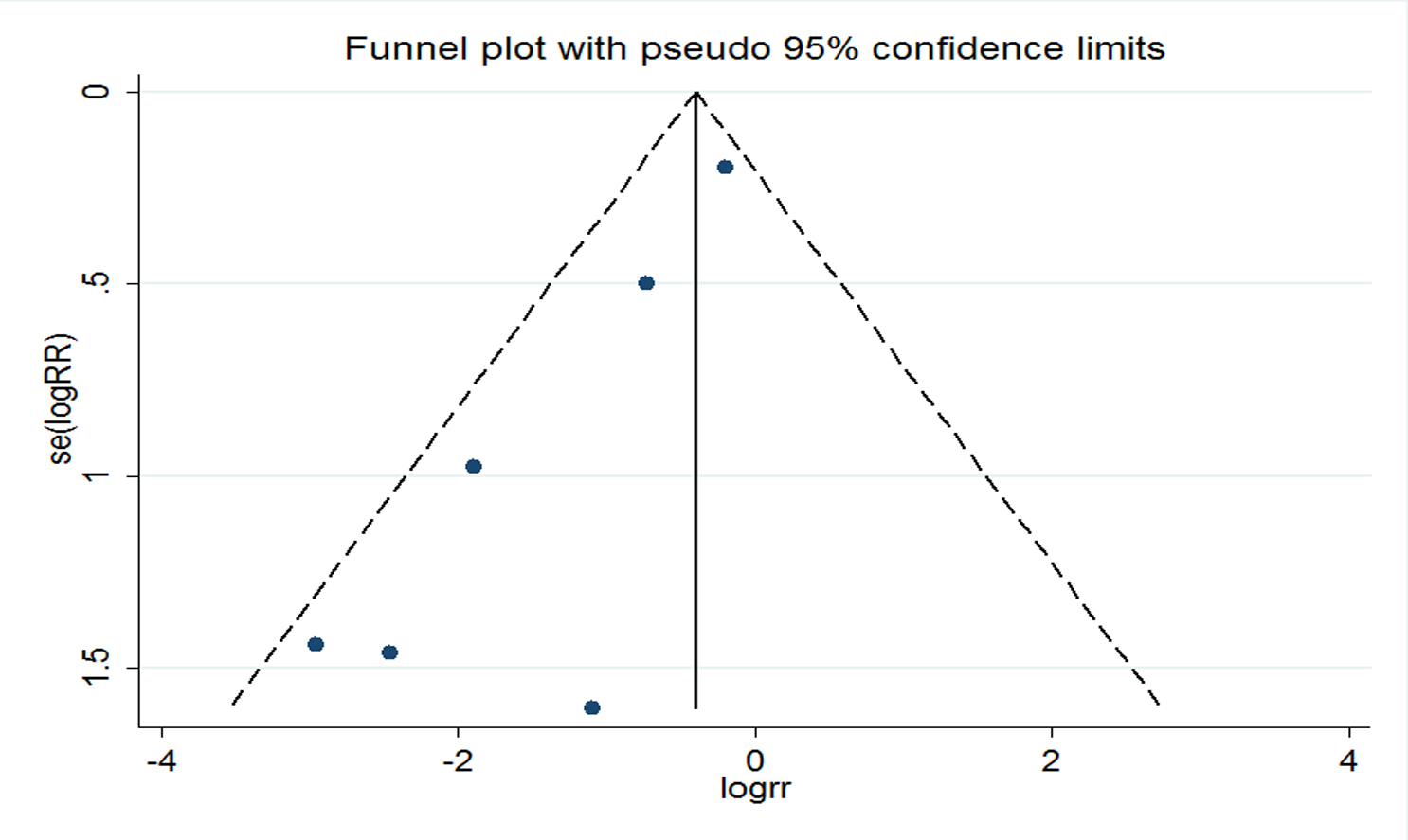

Supplement: Figure S15 — Funnel plot for primary and secondary outcomes. (TIF) [file pone.0096233.s015.tif]

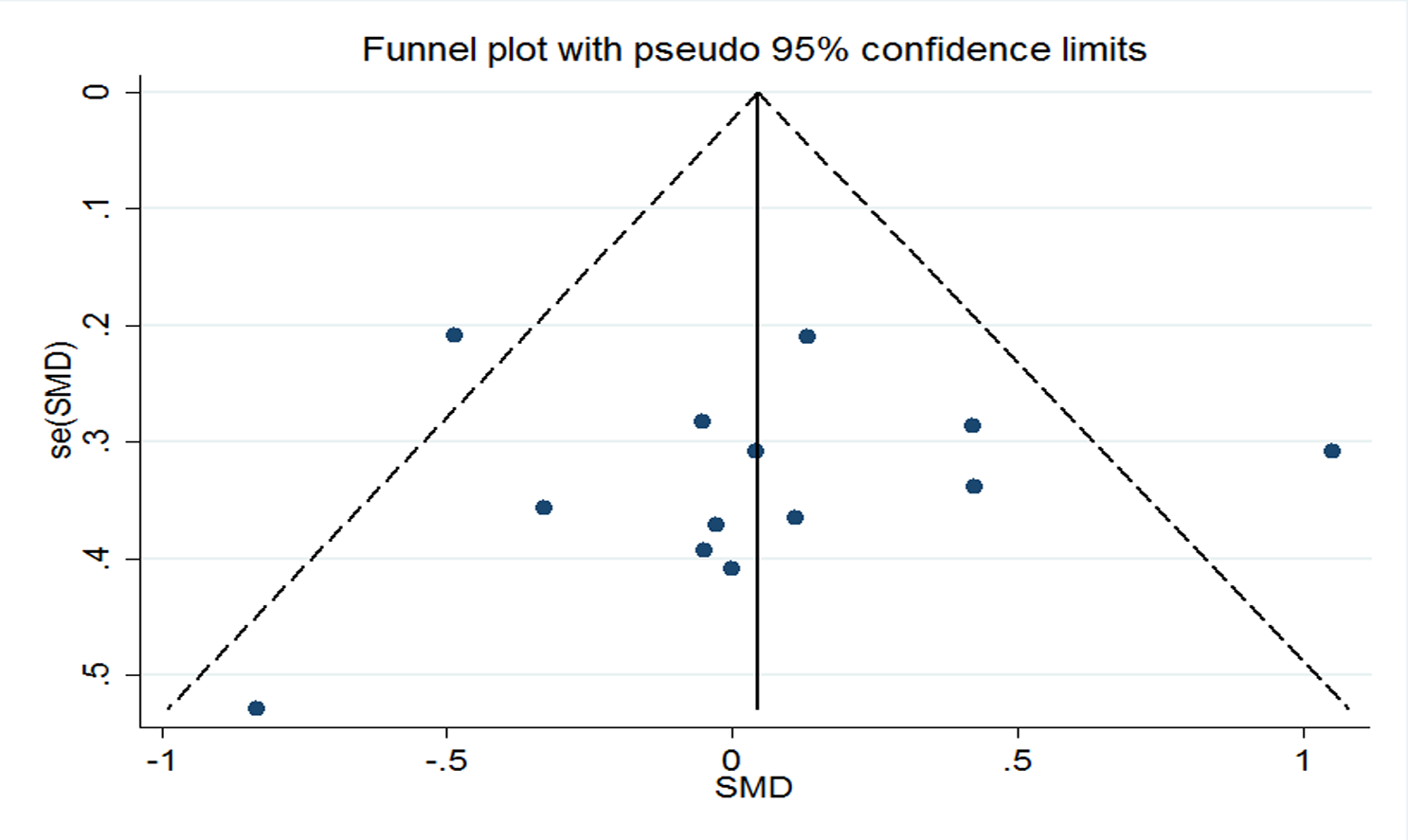

Supplement: Figure S16 — Funnel plot for primary and secondary outcomes. (TIF) [file pone.0096233.s016.tif]

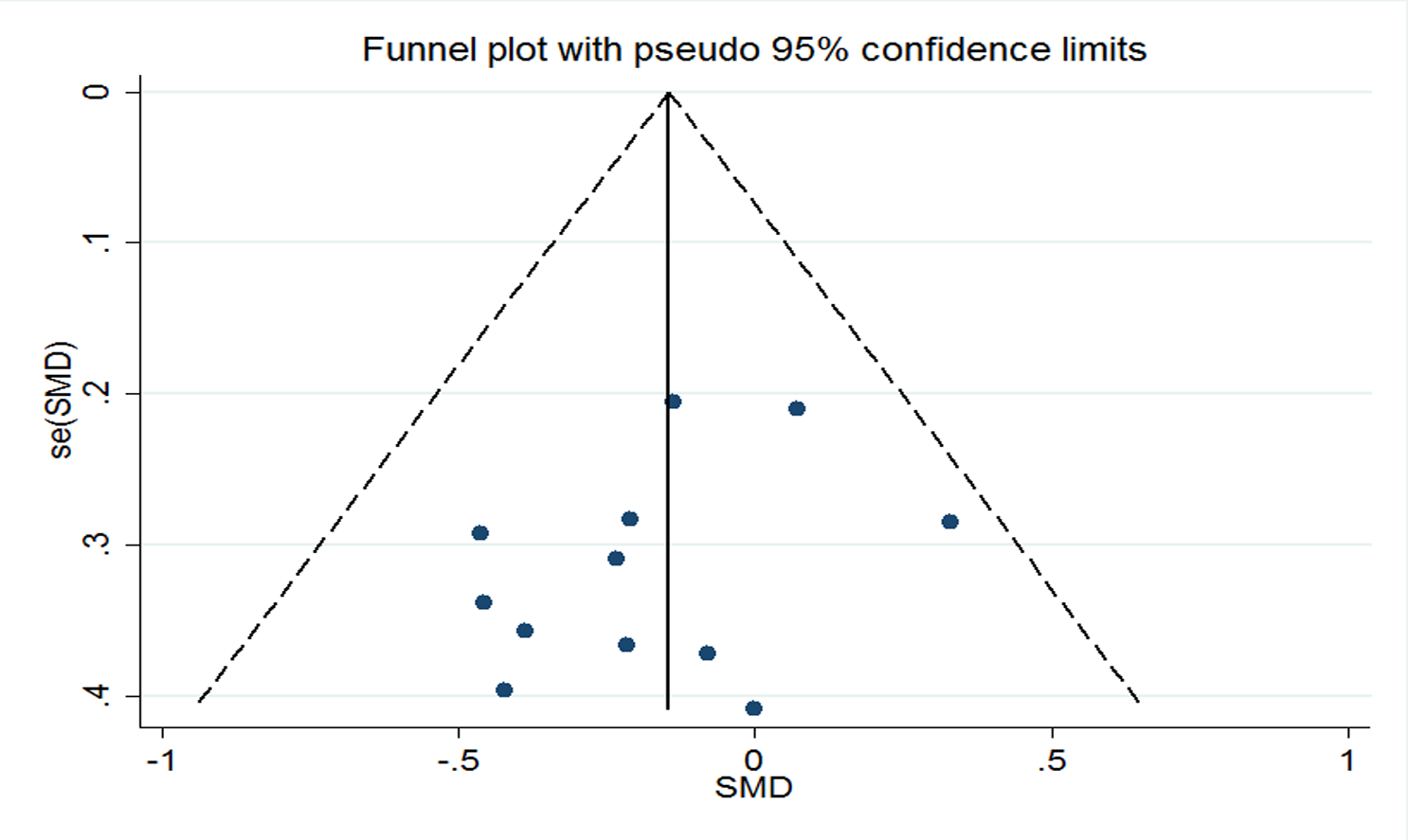

Supplement: Figure S17 — Funnel plot for primary and secondary outcomes. (TIF) [file pone.0096233.s017.tif]

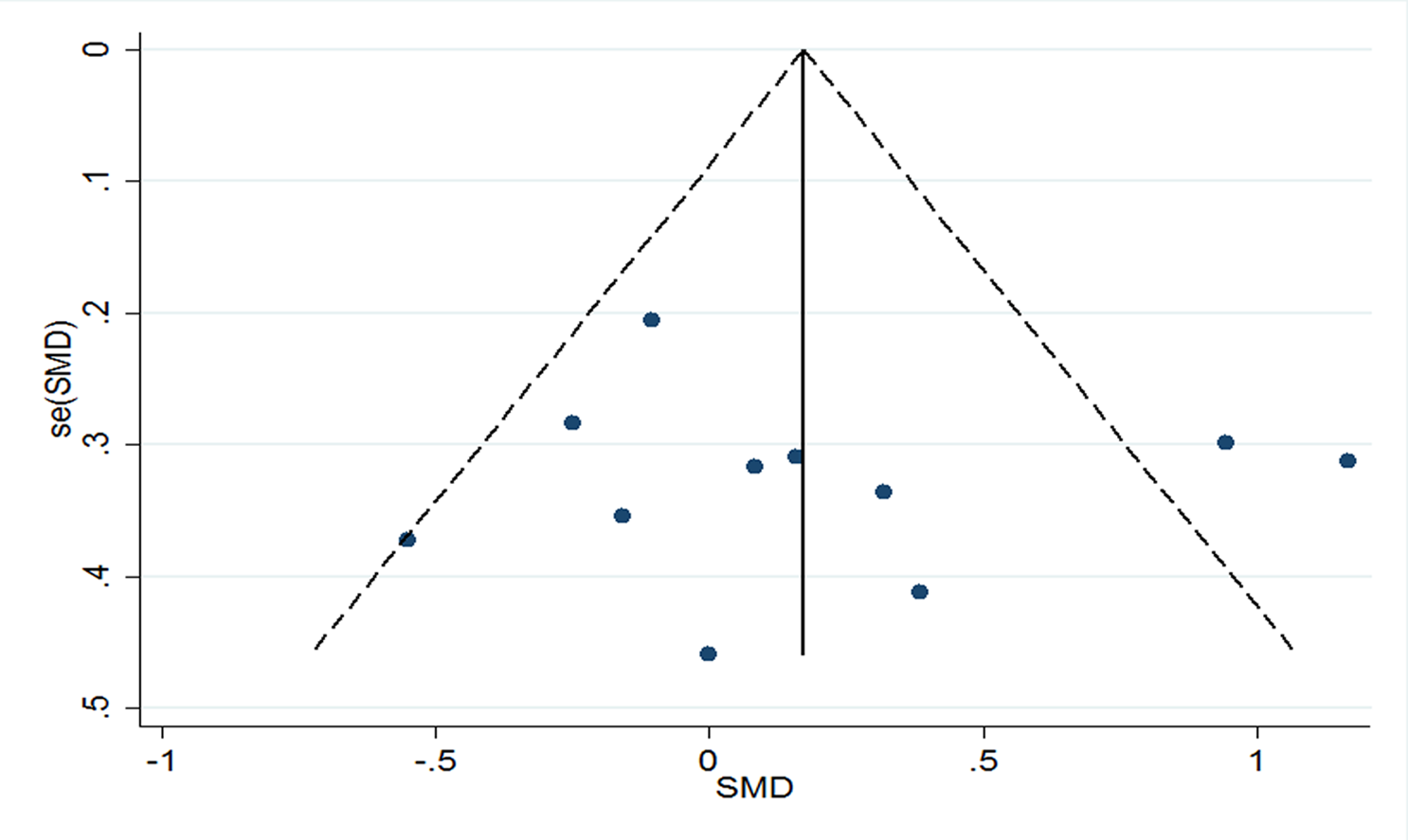

Supplement: Figure S18 — Funnel plot for primary and secondary outcomes. (TIF) [file pone.0096233.s018.tif]
