# Supplementary material for: A Comparison of the Analgesia Efficacy and Side Effects of Paravertebral Compared with Epidural Blockade for Thoracotomy: An Updated Meta-Analysis
Source: PLoS One. 2014 May 5;9(5):e96233. doi: 10.1371/journal.pone.0096233 (PMC4010440; doi:10.1371/journal.pone.0096233)
Supplement: Checklist S1 — PRISMA 2009 Checklist. (DOC) [file pone.0096233.s019.doc]

| **Section/topic** | **#** | **Checklist item** | **Reported on page #** |
| --- | --- | --- | --- |
| **TITLE** | | |  |
| Title | 1 | Identify the report as a systematic review, meta-analysis, or both. | Meta-analysis |
| **ABSTRACT** | | |  |
| Structured summary | 2 | Provide a structured summary including, as applicable: background; objectives; data sources; study eligibility criteria, participants, and interventions; study appraisal and synthesis methods; results; limitations; conclusions and implications of key findings; systematic review registration number. | Objective, Summary of Background, Methods, Result, Conclusions. |
| **INTRODUCTION** | | |  |
| Rationale | 3 | Describe the rationale for the review in the context of what is already known. | epidural analgesia is clearly effective for managing postoperative pain after thoracotomy, it still has limitations and contraindications. |
| Objectives | 4 | Provide an explicit statement of questions being addressed with reference to participants, interventions, comparisons, outcomes, and study design (PICOS). | The current study is an updated meta-analysis comparing the efficacy and adverse effects of PVB and EPI in preventing pain associated with thoracotomy. |
| **METHODS** | | |  |
| Protocol and registration | 5 | Indicate if a review protocol exists, if and where it can be accessed (e.g., Web address), and, if available, provide registration information including registration number. | no |
| Eligibility criteria | 6 | Specify study characteristics (e.g., PICOS, length of follow-up) and report characteristics (e.g., years considered, language, publication status) used as criteria for eligibility, giving rationale. | Randomized controlled trials that compared the PVB and EPI for thoracotomy were included. Studies published only in English were included. |
| Information sources | 7 | Describe all information sources (e.g., databases with dates of coverage, contact with study authors to identify additional studies) in the search and date last searched. | Pubmed, EMBASE, and the Cochrane Library for reports published from 1 January 2006 to 2 February 2013. |
| Search | 8 | Present full electronic search strategy for at least one database, including any limits used, such that it could be repeated. | The following medical subject headings were included: paravertebral, epidural, thoracotomy, and randomized controlled trial. Only studies concerning thoracotomy were allowed and trials regarding breast cancer, and lumbar epidural block were excluded. |
| Study selection | 9 | State the process for selecting studies (i.e., screening, eligibility, included in systematic review, and, if applicable, included in the meta-analysis). | Two reviewers used the pre-specified criteria to screen for relevant titles, abstracts, and full papers. An article was removed if it did not meet the inclusion criteria. If these reviewers reached different final selection decisions, a third reviewer was consulted. |
| Data collection process | 10 | Describe method of data extraction from reports (e.g., piloted forms, independently, in duplicate) and any processes for obtaining and confirming data from investigators. | We extracted the data from the included articles. The definitions of the indicators conformed to those of the original authors. The two reviewers who selected the appropriate studies also extracted the data and evaluated the risk of bias. An arbiter was consulted to reconcile any disagreement. |
| Data items | 11 | List and define all variables for which data were sought (e.g., PICOS, funding sources) and any assumptions and simplifications made. | As the primary outcomes, we defined the analgesic effect in terms of VAS scores at postoperative 4-8 h, 24 h, 48 h, and morphine usage during the first 24 h. Secondary outcomes were the remaining pulmonary complications and urinary retention. |
| Risk of bias in individual studies | 12 | Describe methods used for assessing risk of bias of individual studies (including specification of whether this was done at the study or outcome level), and how this information is to be used in any data synthesis. | We used the Cochrane Handbook V5.0.2 to assess the risk of bias for all articles. The following information was evaluated: random sequence generation, allocation concealment, blinding, incomplete outcome data, selective reporting, and other bias. |
| Summary measures | 13 | State the principal summary measures (e.g., risk ratio, difference in means). | Review Manager Software was used for the meta-analysis. For the continuous variables in the studies included in this meta-analysis (VAS score at postoperative 4-8, 24 and 48 h, and morphine usage at 24 h), used mean difference (MD) and 95% confidence interval (95% CI). For dichotomous variables (pulmonary complications, urinary retention, nausea and vomiting, hypotension, and failed rates of blockage), we used the odds ratio (OR) and 95% CI. |
| Synthesis of results | 14 | Describe the methods of handling data and combining results of studies, if done, including measures of consistency (e.g., I^2^) for each meta-analysis. | Heterogeneity among the studies was evaluated using the I^2^ statistic and chi-squared test. A fixed effects model was used if the heterogeneity test did not reveal a statistical significance (I^2^<50%, p>0.1). Otherwise, we adopted the random effects model. |

Page 1 of 2

| **Section/topic** | **#** | **Checklist item** | **Reported on page #** |
| --- | --- | --- | --- |
| Risk of bias across studies | 15 | Specify any assessment of risk of bias that may affect the cumulative evidence (e.g., publication bias, selective reporting within studies). | Publication bias |
| Additional analyses | 16 | Describe methods of additional analyses (e.g., sensitivity or subgroup analyses, meta-regression), if done, indicating which were pre-specified. | If the heterogeneity was>50%, we performed a sensitivity analysis by sequentially removing each study and reanalyzing the remaining dataset. Also, we analyzed only data that had a low risk of bias. |
| **RESULTS** | | |  |
| Study selection | 17 | Give numbers of studies screened, assessed for eligibility, and included in the review, with reasons for exclusions at each stage, ideally with a flow diagram. | Figure 1. Flow chart of study selection |
| Study characteristics | 18 | For each study, present characteristics for which data were extracted (e.g., study size, PICOS, follow-up period) and provide the citations. | Table 1. Characteristics of included studies |
| Risk of bias within studies | 19 | Present data on risk of bias of each study and, if available, any outcome level assessment (see item 12). | Table 2. Risk of bias assessment of included studies |
| Results of individual studies | 20 | For all outcomes considered (benefits or harms), present, for each study: (a) simple summary data for each intervention group (b) effect estimates and confidence intervals, ideally with a forest plot. | Figure 2. Meta-analyses of postoperative analgesic efficacy of PVB compared with that of EPI  Figure 3. Meta-analyses of adverse side effect of PVB with that of EPI |
| Synthesis of results | 21 | Present results of each meta-analysis done, including confidence intervals and measures of consistency. | Table 3. All detailed results |
| Risk of bias across studies | 22 | Present results of any assessment of risk of bias across studies (see Item 15). | Visual inspection of the funnel plot and Egger’s test for publication bias (Figure 4-21) suggests that there was no evidence of publication bias in VAS scores at postoperative 4-8 h, 24 h, 48 h or for morphine usage, hypotension, rates of failed technique, or pulmonary complications. However, there was publication bias in urinary retention and nausea and vomiting. |
| Additional analysis | 23 | Give results of additional analyses, if done (e.g., sensitivity or subgroup analyses, meta-regression [see Item 16]). | We performed a sensitivity analysis of VAS scores at postoperative 4-8 and 24h. We found that only when Bimston et al. was excluded could heterogeneity be resolved at VAS 4-8h, but the results did not change. The exclusion of Bimston et al. or Richardson et al. resolved the heterogeneity of VAS scores at 24 h, but this also did not change the results. When we analyzed only data from studies with low risk of bias, we found no heterogeneity=0%, but there was still no change in results. |
| **DISCUSSION** | | |  |
| Summary of evidence | 24 | Summarize the main findings including the strength of evidence for each main outcome; consider their relevance to key groups (e.g., healthcare providers, users, and policy makers). | PVB is associated with less urinary retention, postoperative nausea and vomiting, and hypotension. However, we also found that there were no significant differences in pulmonary complications |
| Limitations | 25 | Discuss limitations at study and outcome level (e.g., risk of bias), and at review-level (e.g., incomplete retrieval of identified research, reporting bias). | relatively low quality data with a high risk of bias, surgical placement of the catheter under direct vision must influence the results of side effects because it avoids complications and reduces failure rates, various drug regimens |
| Conclusions | 26 | Provide a general interpretation of the results in the context of other evidence, and implications for future research. | This meta-analysis showed that PVB can provide comparable pain relief to traditional EPI, and may have a better side-effect profile for pain relief after thoracic surgery. Further high-powered randomized trials are to need to determine whether PVB truly offers any advantages over EPI. |
| **FUNDING** | | |  |
| Funding | 27 | Describe sources of funding for the systematic review and other support (e.g., supply of data); role of funders for the systematic review. | National Natural Science Foundation (81270135) and Shanghai Education Committee Key Project (13ZZ024). |

*From:*  Moher D, Liberati A, Tetzlaff J, Altman DG, The PRISMA Group (2009). Preferred Reporting Items for Systematic Reviews and Meta-Analyses: The PRISMA Statement. PLoS Med 6(6): e1000097. doi:10.1371/journal.pmed1000097

For more information, visit: **www.prisma-statement.org**.

Page 2 of 2
