# Supplementary material for: A Comparison of the Analgesia Efficacy and Side Effects of Paravertebral Compared with Epidural Blockade for Thoracotomy: An Updated Meta-Analysis
Source: PLoS One. 2014 May 5;9(5):e96233. doi: 10.1371/journal.pone.0096233 (PMC4010440; doi:10.1371/journal.pone.0096233)
Supplement: Appendix S1 — The Cochrane Library search strategy. (DOC) [file pone.0096233.s020.doc]

Records identified through database searching

(n =48 )

Records excluded by title and abstracts

(n =37)

Full-text articles assessed for eligibility

(n =11 )

Full-text articles excluded, with reasons

No English language (n=4)

Unrelated to the study aims (n=3)

(n = 7 )

Studies included in qualitative synthesis

(n = 4 )

Records excluded by removed duplicates

(n =4)

Studies included in qualitative synthesis

(n = 0)
